# Optimization of a lentivirus-mediated gene therapy targeting HIV-1 RNA to eliminate HIV-1-infected cells

**DOI:** 10.1016/j.omtn.2024.102341

**Published:** 2024-09-16

**Authors:** Amanda B. Buckingham, Sophia Ho, Finlay Knops-Mckim, Carin K. Ingemarsdotter, Andrew M.L. Lever

**Affiliations:** 1 University of Cambridge, Department of Medicine, Level 5 Addenbrooke’s Hospital, Hills Rd, Cambridge CB2 0QQ, UK; 2 Independent Researcher, Cambridge, UK

**Keywords:** MT: Oligonucleotides: Therapies and Applications, HIV-1, RNA *trans*-splicing, herpes simplex virus, HSVtk/GCV, gene therapy, lentiviral vector, shock and kill, latency reversing agents, latent reservoir

## Abstract

Persistence of HIV-1 in cellular reservoirs results in lifelong infection, with cure achieved only in rare cases through ablation of marrow-derived cells. We report on optimization of an approach that could potentially be aimed at eliminating these reservoirs, hijacking the HIV-1 alternative splicing process to functionalize the herpes simplex virus thymidine kinase (HSVtk)/ganciclovir (GCV) cell suicide system through targeted RNA *trans*-splicing at the HIV-1 D4 donor site. AUG1-deficient *HSVtk* therapeutic pre-mRNA was designed to gain an in-frame start codon from HIV-1 *tat1*. D4-targeting lentiviral vectors were produced and used to transduce HIV-1-expressing cells, where *trans*-spliced HIV-1 *tat*/*HSVtk* mRNA was successfully detected. However, translation of catalytically active HSVtk polypeptides from internal AUGs in *HSVtk*_ΔAUG1_ caused GCV-mediated cytotoxicity in uninfected cells. Modifying these sites in the D4 opt 2 lentiviral vector effectively mitigated this major off-target effect. Promoter choice was optimized for increased transgene expression. Affinity for HIV-1 RNA predicted *in silico* correlated with the propensity of opt 2 payloads to induce HIV-1 RNA *trans*-splicing and killing of HIV-1-expressing cells with no significant effect on uninfected cells. Following latency reversing agent (LRA) optimization and treatment, 45% of lymphocytes in an HIV-1-infected latency model could be eliminated with D4 opt 2/GCV. Further development would be warranted to exploit this approach.

## Graphical abstract

**Figure:**
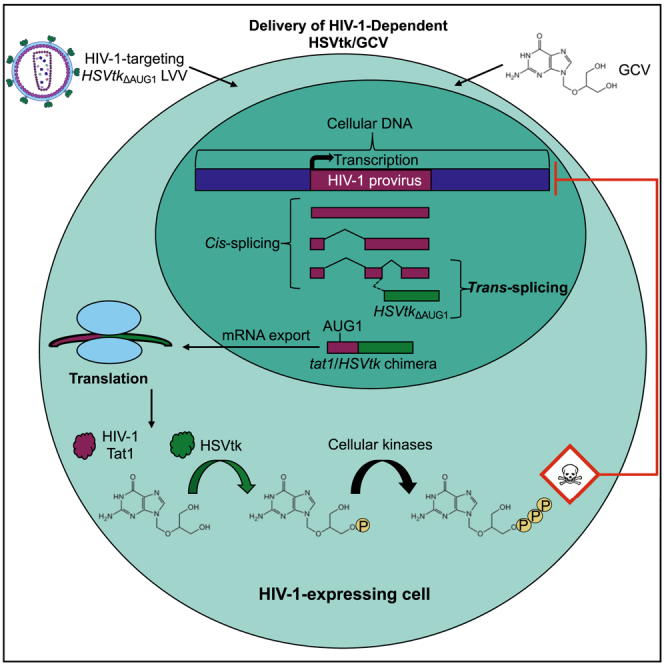

## Teaser

Buckingham and colleagues improved the potential of an HIV-1 RNA-targeted cell suicide system to antagonize HIV-1-infected cells while sparing healthy cells. The lead therapeutic candidate established from their study in immortalized cell lines would merit further investigation and development given the lifelong persistence of HIV-1 in cellular reservoirs.

## Introduction

In 2023, approximately 77% of the 39.9 million people living with human immunodeficiency virus 1 (HIV-1) accessed antiretroviral therapy (ART),^1^ which has transformed a once-terminal prognosis into a manageable, albeit chronic, condition. Drug-resistant viral variants are a growing problem and substantially increase the likelihood of treatment failure,^2^ necessitating the development of treatments against novel targets in HIV-1 replication. In individuals considered to be virologically suppressed, inflammation does not fully resolve and increases the risk of developing cardiovascular, neurocognitive, kidney, and liver diseases.^3^ This is due in part to ongoing low-level viremia (approximately 1–5 HIV-1 RNA copies/mL in plasma) fueled by cellular reservoirs carrying HIV-1 in a reversible state of latency.^4^^,^^5^

One experimental approach to target and clear the latent HIV-1 reservoir is known as “shock and kill,” which aims to induce transcription from dormant HIV-1 proviruses such that viral gene products will prove fatal to infected cells or enable their recognition and eradication by cytotoxic immune cells.^6^ Clinical trials have demonstrated that disruption of latency is feasible in people living with HIV-1 (PLWH), with a marked, albeit transient, increase in HIV-1 RNA levels following *in vivo* administration of histone deacetylase inhibitors such as vorinostat^7^ and romidepsin^8^ and Akt activators such as disulfiram.^9^ However, as treatment with such latency reversing agents (LRAs) has yet to translate into robust reductions in latent reservoir size,^5^^,^^10^ it has been proposed that additional therapeutic interventions will be needed to specifically kill cells harboring HIV-1 reactivated from latency.^10^^,^^11^ Strategies currently under investigation include enhancing the immune response (e.g., with toll-like receptor agonists or checkpoint blockade)^12^ and identifying pro-apoptotic compounds (e.g., Bcl-2 antagonists) that preferentially kill chronically HIV-1-infected cells, which are skewed toward a pro-survival phenotype.^11^^,^^13^

Previously, we found that HIV-1 alternative splicing—a critical yet clinically unexploited aspect of viral replication^14^^,^^15^—can be hijacked to functionalize the herpes simplex virus thymidine kinase (HSVtk)/ganciclovir (GCV) cell suicide system (CSS), resulting in the killing of HIV-1-expressing cells.^16^ This strategy is unique among other HIV-1-dependent HSVtk/GCV CSSs in development^17^ in its ability to target HIV-1 at an RNA level,^16^ which could be of particular use in the shock-and-kill setting. The lead *trans*-splicing candidate we identified, known as BD1-D4, encodes an AUG1-deficient *HSVtk* pre-mRNA that targets the HIV-1 D4 donor splice site for RNA *trans*-splicing by 3′ exon replacement, in which the first exon of *tat* is joined with *HSVtk*_ΔAUG1_ in lieu of *tat* exon 2.^16^ In the resultant chimeric mRNA, translation is directed from *tat* exon 1 AUG1 in frame with the *HSVtk* coding sequence, allowing for the synthesis of the near-full-length cell suicide enzyme (nflHSVtk) upon p2A cleavage.^16^

To better antagonize chronic HIV-1 infection in PLWH, in the present study we investigated a well-established^18^ lentiviral vector (LVV)^19^^,^^20^-based system for stable expression of the BD1-D4 transgene. We optimized LVV production, identified and subsequently modified sites in the therapeutic payload that threatened the HIV-1-dependency of our approach, refined our choice of promoter for optimal payload expression, and demonstrated through a combination of *in vitro* and *in silico* assays that our lead therapeutic candidate D4 opt 2 selectively reduced the viability of HIV-1-expressing cell lines with no significant effect on uninfected cells. We hypothesized that chronically HIV-1-infected cells may be susceptible to our HIV-1-RNA-targeted CSS following stimulation of HIV-1 expression with LRAs, and found that this approach could be used to antagonize the viability of a T cell line model of HIV-1 latency. Optimization of LRA choice heightened the killing effect. Collectively, our results suggest that targeting HIV-1 RNA alternative splicing with an LVV-mediated, HIV-1-dependent CSS has the potential to increase the vulnerability of HIV-1-harboring cells and enhance the “kill” in shock and kill. Further study and development would be warranted in primary cell models of HIV-1 infection.

## Results

### Validation of HIV-1 RNA-targeting lentiviral vectors in an HIV-1-expressing T cell line

**Figure 1:**
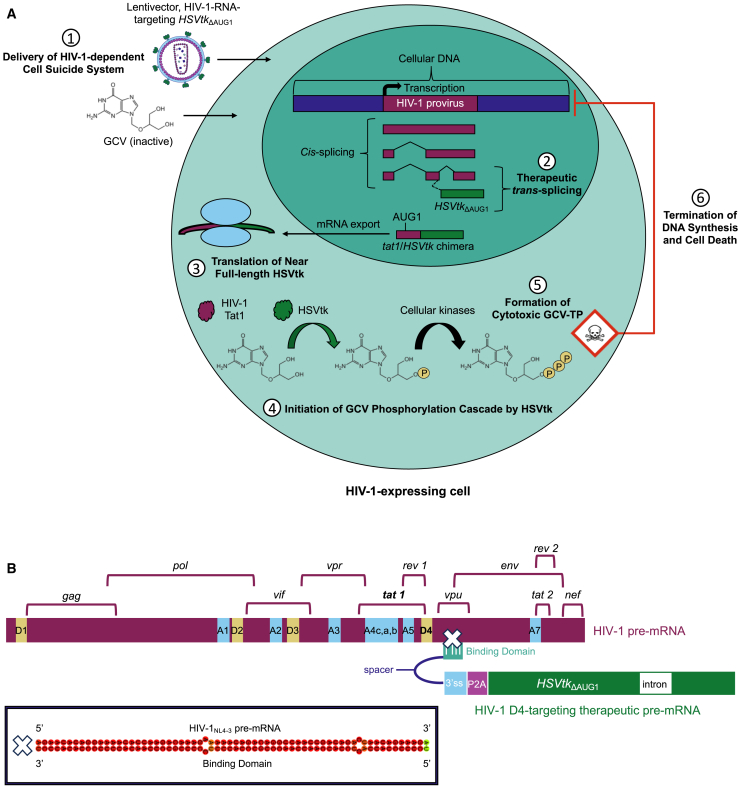
A lentivirus-mediated gene therapy targeting HIV-1 RNA to eliminate HIV-1-expressing cells (A) Schematic for delivery and activation of HIV-1-dependent HSVtk/GCV CSS. Following LVV transduction, the HIV-1-targeting *HSVtk*_ΔAUG1_ payload is expressed at the RNA level and subsequently functionalized in HIV-1-expressing cells through an RNA *trans*-splicing reaction with the HIV-1 *tat 1* exon. Near-full-length HSVtk is translated from the chimeric HIV-1 *tat*/*HSVtk* mRNA and phosphorylates the prodrug GCV, initiating a phosphorylation cascade by cellular kinases that culminates in GCV-TP, a cytotoxic metabolite that induces cell death through DNA damage. (B) Schematic by which HIV-1 pre-mRNA is targeted for therapeutic *trans*-splicing. The therapeutic pre-mRNA localizes to HIV-1 pre-mRNA by means of a complementary binding domain. This positions the therapeutic splice acceptor (3′ss) for RNA *trans*-splicing with the HIV-1 D4 splice donor, which is proximal to the binding domain target sequence in HIV-1 *vpu*. The approximate locations of HIV-1 open reading frames and splice sites are annotated above and within the pre-mRNA, respectively. The HIV-1_NL4-3_:binding domain RNA duplex is adapted from Figure 3A, with the spacer region requisite for RNA secondary structure modeling digitally removed. ss, splice site; D, donor (5′ss); A, acceptor (3′ss). (A and B) Diagrams are not drawn to scale.

**Figure 2:**
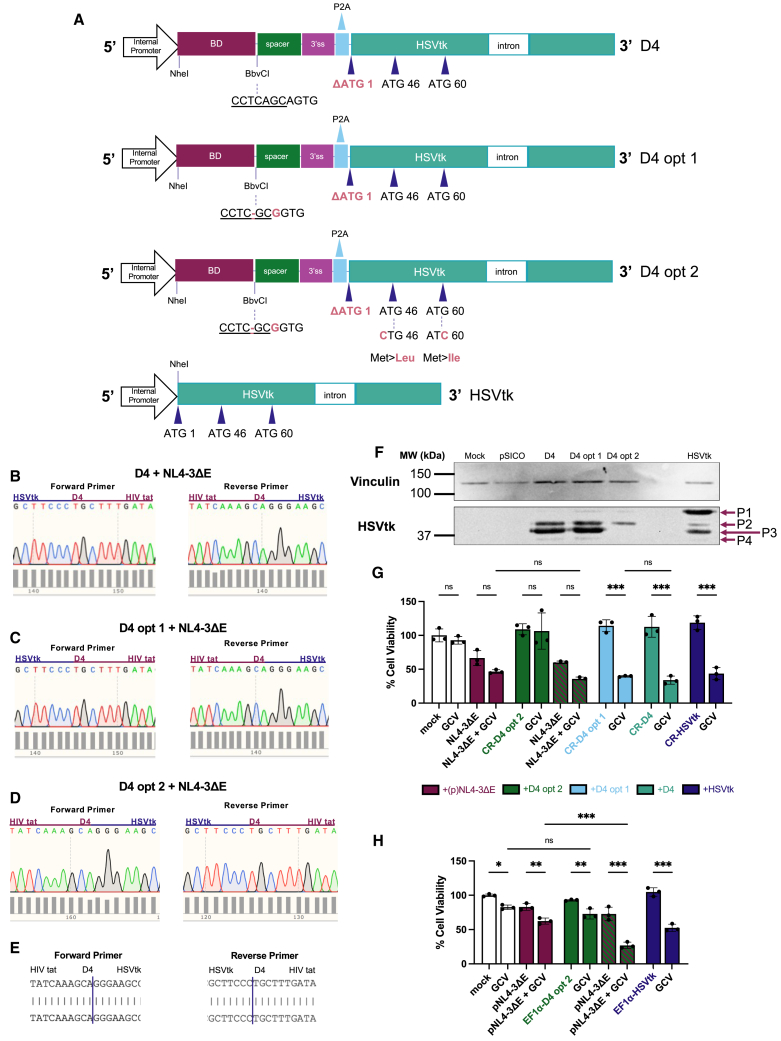
Optimization of HSVtk translational initiation from HIV-1 RNA-targeting LVVs is necessary to restrict HSVtk/GCV-mediated killing to HIV-1-expressing cells (A) Maps of HIV-1 RNA-targeting *trans*-splicing cassettes D4, D4 opt 1, and D4 opt 2 illustrating modifications made to *HSVtk* and *Bbv*CI to alter downstream translational initiation sites and an additional predicted splice acceptor site, respectively. The full-length *HSVtk*-positive control cassette is shown for comparison. BD, binding domain. 3’ss, splice acceptor site. (B–E) Sanger sequencing confirmation of *trans*-spliced HIV-1/*HSVtk* RNA in HIV-1-expressing Jurkat T cells following delivery of (B) D4, (C) D4 opt 1, or (D) D4 opt 2 therapeutic LVV. Chromatogram snapshots depict the HIV-1/*HSVtk* splice junction. Refer to Figure S4 for experimental details. In brief, *trans*-spliced products were amplified from Jurkat RNA by RT-PCR and cloned into TOPO plasmids (where they could be inserted in either orientation) for sequencing. M13 primers were used to read from the TOPO backbone into the insert. (E) Representative BLAST alignment between the predicted splice junction and the amplified *trans*-spliced product from HIV-1_NL4-3ΔE_-expressing Jurkat cells transduced with D4 opt 2. (F) Anti-HSVtk western blot of polypeptide expression (P1, 43 kDa; P2, 40.4 kDa; P3, 39.8 kDa; P4, 37.0 kDa^23^) from therapeutic constructs D4, D4 opt 1, and D4 opt 2 in the absence of HIV-1, with anti-vinculin (124 kDa) loading control. Full-length *HSVtk* construct (HSVtk) and transfer plasmid backbone (pSico) used as positive and negative controls for *HSVtk* expression, respectively. MW, molecular weight. (G and H) Viability screens in HIV-1-expressing and uninfected cells. (G) 5 × 10^3^ Jurkat T cells/well or (H) 2 × 10^4^ HEK293T cells/well were seeded on day 1, (G) transduced with HIV-1_NL4-3ΔE_ (MOI = 6) or (H) transfected with 100 ng HIV-1_NL4-3ΔE_ plasmid (pNL4-3ΔE) on day 2, transduced with (G) CkRhsp (CR)-driven LVV panel (MOI = 14) or (H) EF1α-driven LVV panel (MOI = 2) on day 3, treated with 50 μM GCV doses on days 4 and 5, and subjected to MTT cell viability assay on day 8. Mock treatments performed with media only. Data presented as mean with SD (*N* = 3 wells/condition). ∗*p* < 0.05, ∗∗*p* < 0.01, ∗∗∗*p* < 0.001; one-way ANOVA with Tukey’s multiple comparisons test.

The BD1-D4 (henceforth known as D4) RNA *trans*-splicing construct described by Ingemarsdotter et al.,^16^ encoding a defective cell suicide gene (*HSVtk*_ΔAUG1_) functionalized by targeted 3′ exon replacement of HIV-1 *tat* exon 2, was developed into an anti-HIV-1 gene therapy candidate delivered by the self-inactivating^20^ third-generation^19^ lentiviral vector system (Figure 1). CkRhsp (CR), constructed with reference to the chimeric HIV-1 Tat-inducible promoter described by Farazmandfar et al.^21^ (section “Construction of CkRhsp promoter”) was initially used to drive transgene expression. *Trans*-splicing cassettes (D4 and two modified versions, D4 opt 1 and D4 opt 2) and the full-length *HSVtk* positive control cassette (HSVtk) (Figure 2A) were cloned into the lentiviral transfer plasmid pSico (Addgene plasmid #11578) to enable packaging into lentiviral particles (section “Generation of RNA trans-splicing lentiviral transfer plasmids”). Lentiviruses were pseudotyped with VSV-G for enhanced particle stability and broad cell tropism^22^ to facilitate *in vitro* proof-of-principle study.

We estimated infectious titer by qPCR (section “Titration of infectious lentiviral particles by qPCR”), based on the average number of lentiviral cDNA copies per cell (vector copy number [VCN]) following transduction of Jurkat T lymphocytes (karyotyped in Figure S1) at set volumes of lentivirus stock.^24^^,^^25^^,^^26^ With this approach, 1 transducing unit (TU) = 1 lentiviral cDNA (i.e., reverse transcript). However, when we initially produced LVVs by transient transfection (Supplemental methods), we found that transfer plasmid predominated over LVV cDNA in transduced cells (Figure S2), inflating infectious titer estimations (Figure S3D). Increasing the scale of production and including an additional step to digest DNA (Figure S3A) in the preparations (see Figure S3B for workflow) effectively controlled plasmid carryover based on qPCR (Figure S3C) and resulted in a higher lentiviral particle yield based on ELISA (Figure S3D). We used this optimized methodology to produce all further therapeutic/control LVV and HIV-1_NL4-3ΔE_ stocks. Therapeutic LVV preparations were of high infectious titer^27^ (∼1 × 10^8^ TU/mL) based on qPCR and had good packaging efficiency (Figure S3E): on average, the proportion of infectious to total particles was 1:1,150 (Figure S3E), close to the established range (1:100 to 1:1,000).^28^

Lentiviruses were validated in Jurkat T cells (Figure S4). Following lentiviral transduction and analysis of cellular DNA by qPCR, we found that VCN was consistent with the intended concentration (Figures S4A, S4B, S4D, and S4E), an expected outcome from using the same cell line for titration. Transgene expression from therapeutic and positive control LVV was next confirmed by RT-qPCR on RNA from transduced Jurkat T cells (Figures S4C and S4F), with the amplicon sequence positioned within *HSVtk*^29^ (Figure S4G) for detection of all potential mRNA classes (AUG1-deficient or *trans*-spliced *HSVtk* for therapeutic LVVs, or full-length *HSVtk* for the positive control). Expression of HIV-1_NL4-3ΔE_ at the RNA level was confirmed by RT-qPCR for *tat* exon 1 (Figures S4C and S4F), onto which the HIV-1-targeting therapeutic RNA payload should *trans-*splice.

We next used RT-PCR to assay for chimeric HIV-1 *tat*/*HSVtk* mRNAs produced through *trans*-splicing in Jurkat T cells,^16^^,^^30^ with primers positioned for amplification of the splice junction (Figure S4H). Putative *trans*-spliced RT-PCR products (142 bp) could be distinguished by gel electrophoresis from HIV-1-expressing Jurkat cells transduced with D4 (Figure S4I), D4 opt 1 or D4 opt 2 (Figure S4J) and were inserted into TOPO (Invitrogen) plasmid vectors (Figures S4K–S4P) for Sanger sequencing (Figures 2B–2D). By BLAST alignment, there was 100% shared nucleotide identity with the predicted *trans*-spliced HIV-1/*HSVtk* sequence (Figure 2E), thus validating that *HSVtk*_ΔAUG1_ delivered to HIV-1-expressing T cells by our panel of therapeutic LVV could *trans*-splice onto HIV-1 *tat* exon 1 to gain an upstream, in-frame translational initiation codon.

Primer dimers and nonspecific amplicons from the RT-PCR also were cloned into TOPO vectors for characterization by Sanger sequencing and BLAST analysis. Through this work, we identified one instance of off-target therapeutic *trans*-splicing between CkRhsp-directed D4 opt 2 and a cellular RNA (Figure S5), though the chimeric sequence was close to the limit of detection (Figure S4J, boxed in red) and did not reoccur. No significant off-target killing was observed from this LVV in the absence of HIV (Figure 2G). Based on BLAST analysis, we found no evidence to suggest that the cellular RNA had been specifically targeted, suggesting that *trans*-splicing had occurred stochastically. Previous studies of lentiviral gene therapy have also detected aberrant *trans*-splicing between human and transgenic pre-mRNAs, most notably at the site of proviral integration.^31^^,^^32^^,^^33^

### Optimization of *HSVtk*_ΔAUG1_ translational initiation prevents HIV-1-independent killing

Having confirmed that the therapeutic LVVs in our panel could induce HIV-1 RNA *trans*-splicing in HIV-1-expressing T cells as intended, we also wanted to explore and mitigate their potential to induce reoccurring off-target effects that could be harmful to healthy cells. *In silico* predictions and previously published reports^23^^,^^34^^,^^35^^,^^36^^,^^37^ suggested that there may be sequences in the D4 *trans*-splicing cassette from Ingemarsdotter et al.^16^ that could promote HIV-1-independent cell death unless otherwise altered. One such sequence was an additional putative splice acceptor site within *Bbv*CI and adjacent nucleotides, which we had modified in both the D4 opt 1 and D4 opt 2 cassettes (Figure 2A) to better constrain *trans*-splicing to our designated splice acceptor.

The D4 opt 2 cassette had two further modifications, which were made to better safeguard the dependence of HSVtk activity on the AUG1 gained from *trans*-splicing with HIV-1. Although full-length HSVtk (P1) is the dominant species when AUG1 is intact,^35^^,^^37^ AUG1 also can be bypassed to allow for N-terminally truncated polypeptides to be expressed from alternative in-frame start codons downstream in *HSVtk*,^23^^,^^34^^,^^35^^,^^36^^,^^37^ which we observed in uninfected HEK293T transfected with the positive control HSVtk construct (Figure 2F). Deletion of AUG1 has been reported to promote higher expression levels of the alternative polypeptides,^37^ which we observed following transfection of uninfected HEK293T with D4 and D4 opt 1 *trans*-splicing cassettes (Figure 2F). P2, P3, and a low quantity of P4 could be detected. To our knowledge, no study has investigated the functionality of each HSVtk species in isolation; however, in a report by Ellison and Bishop, proliferation of cells that predominantly expressed P3 or the full-length product P1 was potently suppressed in the presence of GCV, leading the authors to conclude that the two had similar levels of activity.^23^ We hypothesized that P3 expression from the D4 or D4 opt 1 *trans*-splicing cassettes could cause GCV-mediated cell death in the absence of HIV-1. To address this concern, we made an A>C substitution in AUG46—the established translational initiation site for P3^23^—to create the D4 opt 2 cassette (Figure 2A). For D4 opt 2, we also made a G>C substitution to alter AUG60 (Figure 2A), the only other established alternative start codon in *HSVtk* and the reported initiation site of the minor P4 species.^23^ In uninfected HEK293T cells transfected with D4 opt 2, the sole HSVtk polypeptide expressed was P2 (Figure 2F), initiated from an unidentified non-AUG site(s) between AUG1 and AUG46 and determined by Ellison and Bishop to have very modest tk activity.^23^

We next assessed if the sequence alterations we had made across our therapeutic LVV panel would result in D4, D4 opt 1, and D4 opt 2 LVVs having differing effects on the viability of healthy cells with GCV. In the absence of HIV-1, we transduced Jurkat T cells with our therapeutic LVV panel and observed an increase in GCV-mediated cell death from D4 and D4 opt 1 but not D4 opt 2 with increasing MOI (data not shown). At an MOI of 14, the highest concentration tested, transduction with D4 or D4 opt 1 resulted in a highly significant reduction in viability when Jurkat cells were additionally treated with GCV: 113% to 34.0% for D4 and 114% to 39.8% for D4 opt 1 (Figure 2G). D4 and D4 opt 1 LVV killed GCV-treated Jurkat cells as effectively as the full-length HSVtk positive control LVV, which in combination with GCV reduced viability to 43.5% (Figure 2G). In contrast, there was no significant difference in the viability of D4 opt 2-transduced Jurkat cells with (106%) or without (109%) the GCV substrate for functional HSVtk (Figure 2G), confirming that there was no significant catalytically active enzyme expressed from D4 opt 2 in the absence of HIV-1.

D4 opt 1 differs solely from D4 in the *Bbv*CI modification in the former and solely from D4 opt 2 in the AUG modifications in the latter (Figure 2A). As there was no significant difference in the viability of GCV-treated Jurkat cells transduced with either D4 or D4 opt 1 (Figure 2G), we determined that the *Bbv*CI modification had little effect on HIV-1-independent killing. As the effect of D4 opt 1 LVV markedly diverged from that of D4 opt 2 LVV in GCV-treated cells, we concluded that unchecked alternative AUGs in *HSVtk* were instead the principal cause of off-target toxicity (Figure 2G). This conclusion is supported by the prominence of P3—determined by Ellison and Bishop to induce a level of GCV sensitivity equivalent to the full-length product^23^—in uninfected cells expressing D4 opt 1, in contrast to the complete absence of P3 in uninfected cells expressing D4 opt 2 (Figure 2F). This demonstrates that modification of alternative *HSVtk* AUGs in our HIV-1 RNA-targeted *trans*-splicing cassette was critical to avoid HSVtk/GCV activity in healthy cells, which undermined the therapeutic potential of D4 and D4 opt 1.

In parallel, we investigated whether the modifications made to the *trans*-splicing cassette would still allow for D4 opt 2 to induce the death of HIV-1-expressing cells with GCV. When Jurkat cells were co-transduced with HIV-1_NL4-3ΔE_ and D4 opt 2, viability was reduced from 59.9% to 35.7% in the presence of GCV, though the difference was not statistically significant (Figure 2G). However, the innate toxicity of HIV-1_NL4-3ΔE_ in Jurkat T cells may have made HIV-1-dependent activity from the D4 opt 2 CSS more difficult to resolve (Figure 2G). Due to its potential to eliminate a subset of HIV-1-expressing cells without inducing non-selective cell death, D4 opt 2 was determined to be the lead therapeutic candidate and was subject to further optimization for enhanced on-target killing.

### Optimized D4 opt 2 LVV kills HIV-1-expressing cells with GCV

We focused on enhancing payload expression from D4 opt 2. This strategy was undertaken because the initial promoter we constructed for transgene expression, CkRhsp, did not function as expected. CkRhsp as described by Farazmandfar et al.^21^ is HIV-1 Tat inducible; however, the version of CkRhsp we constructed was leaky to the extent that Jurkat T cells transduced with therapeutic LVV in the presence or absence of Tat-expressing HIV-1_NL4-3ΔE_ exhibited similar RNA payload levels (Figures S4C and S4F). We investigated the effect of HIV-1 Tat on our CkRhsp promoter in isolation using two different *in vitro* systems and observed neither an induction nor enhancement in transgene expression at the RNA level based on RT-qPCR (Figure S6). We concluded that our CkRhsp promoter functioned independently of HIV-1 Tat and was thus distinct from that described by Farazmandfar et al.^21^ We hypothesize that differences in the relative position of each domain in our CkRhsp promoter compared to that described in Farazmandfar et al.^21^ may have caused the regulatory switch imposed by the hybrid chicken *β-actin*/HIV-1 R region to become disconnected from *hsp70*-directed transgene expression.

We compared the activity of our CkRhsp promoter to that of the well-established human EF1α promoter^38^ to determine if the latter resulted in superior levels of transgene expression. We replaced CkRhsp with EF1α (section “Replacement of CkRhsp with EF1α promoter”) in the D4 opt 2 and HSVtk lentiviral transfer plasmids and produced EF1α-directed LVV for comparison against the original panel. At a comparable VCN (Figure S7A), we found that payload levels were 4.4 times higher from EF1α-directed D4 opt 2 compared to CkRhsp-directed D4 opt 2 in transduced Jurkat T cells, a highly significant difference (Figure S7B). In addition, when the full-length *HSVtk* cassette was placed under control of EF1α in lieu of CkRhsp, we observed an enhancement in HSVtk/GCV CSS activity (Figure S7C). We hypothesized that replacement of CkRhsp with EF1α as the transgene promoter would improve the therapeutic potential of D4 opt 2 by increasing the impact of each successful transduction event with higher RNA payload levels.

Although Jurkat T cells were sensitive to transduction with VSV-G pseudotyped HIV-1_NL4-3ΔE_ LVV particles (Figure 2G), we found that delivery of HIV-1_NL4-3ΔE_ by a plasmid vector was better tolerated in HEK293T (Figure 2H), allowing for a clearer differential between the toxicity inherent to HIV-1 and the selective toxicity we sought to induce with our HIV-1 RNA-targeted CSS. At an MOI of 2, EF1α-directed D4 opt 2 LVV significantly reduced the viability of HIV-1_NL4-3ΔE_-expressing HEK293T cells from 72.9% to 27.1% in the presence of GCV (Figure 2H). Although the combination of HIV-1_NL4-3ΔE_ and GCV did have an impact on viability, the score (62.4%) was significantly higher than that of HIV-1-expressing HEK293T treated with all components of the HIV-1 RNA-targeted CSS (27.1%) (Figure 2H). In contrast, the viability of healthy HEK293T cells subjected to both EF1α-directed D4 opt 2 and GCV did not significantly differ from that of cells treated with GCV alone (Figure 2H), suggesting that our CSS had little activity in the absence of HIV-1. We concluded that EF1α-directed D4 opt 2, optimized for higher HIV-1 RNA-targeting payload levels and better safeguards against HIV-1-independent HSVtk/GCV activity, constituted a major improvement in the potency and selectivity of our anti-HIV-1 gene therapy.

### HIV-1 RNA *trans*-splicing at the D4 donor site drives HIV-1-expressing cell elimination and is potentiated by the interaction between HIV-1 target and binding domain RNAs

To facilitate HIV-1 *trans*-splicing at D4 for translation of nflHSVtk, the binding domain in therapeutic pre-mRNA was designed to hybridize to a region in *vpu* proximal to the target HIV-1 splice donor (Figure 1B).^16^ To further interrogate the HIV-1 selectivity of our lead therapeutic candidate D4 opt 2, we sought to create a scrambled binding domain version that would be unable to target HIV-1 D4 for completion of its *HSVtk* message, hypothesizing that such a pre-mRNA would fail to induce GCV-mediated cytotoxicity in HIV-1-expressing cells.

**Figure 3:**
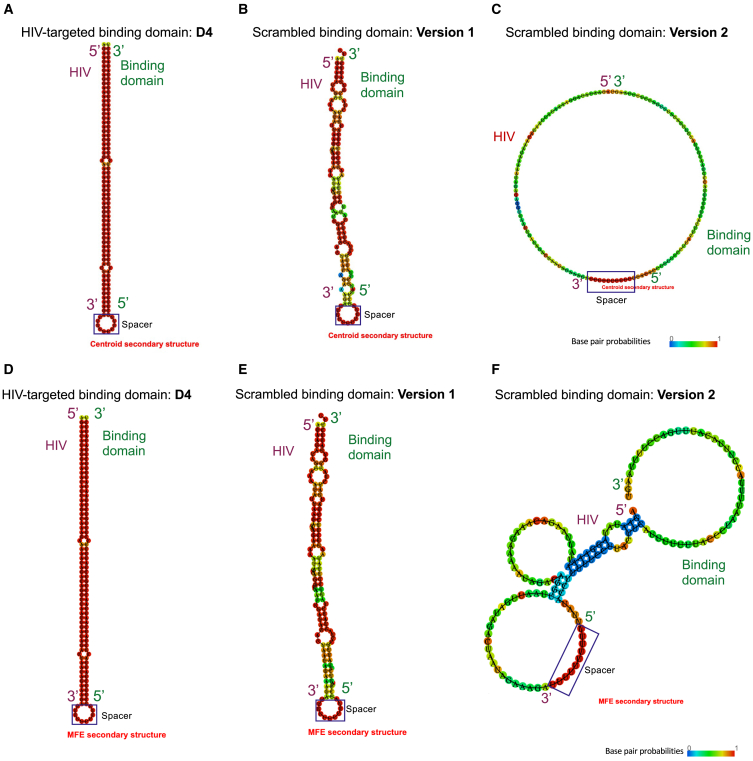
*In silico* RNA secondary structure modeling of the HIV-1 RNA-targeting potential of RNA binding domains (A–C) Centroid secondary structure predictions. Minimum free energy (MFE): (A) −84.50 kcal/mol, (B) −11.20 kcal/mol, (C) 0.00 kcal/mol (D–F) MFE secondary structure predictions. MFE: (D) −84.50 kcal/mol, (E) −16.00 kcal/mol, (F) −6.28 kcal/mol. Duplexes formed between HIV-1_NL4-3_ and either the (A and D) HIV-1 D4-targeting or (B and E) Scramble Version 1 (V1) binding domains. In comparison, HIV-1_NL4-3_ and (C and F) the Scramble Version 2 (V2) binding domain were predicted to make no or reduced contact.

We confirmed that our candidate scrambled binding domain (Scramble V1) shared no identity with the target HIV-1 sequence based on primary structure BLAST analyses (data not shown); however, as part of the therapeutic pre-mRNA molecule, the binding domain would have the potential to make a more varied range of interactions with HIV-1 pre-mRNA, including non-consecutive and noncanonical (i.e., G-U) base pairing.^39^ To account for these interactions, we followed the methodology of D’Souza et al.^40^ to model secondary structure formation between the two RNAs *in silico*, using the Vienna RNAfold Webserver (Figure 3). Unexpectedly, we found that a duplex was predicted to form between Scramble V1 and HIV-1 target RNAs (Figures 3B and 3E), similar to the interaction between the D4 binding domain and HIV-1 target RNAs (Figures 3A and 3D). Based on the minimum free energy (MFE) of the centroid structure—best representative of the range of structure predictions^41^—HIV-1:Scramble V1 duplex stability was lower (−11.20 kcal/mol; Figure 3B) than that of the HIV-1:D4 duplex (−84.50 kcal/mol; Figure 3A), with similar results when the structures with the lowest MFE were compared (−16.00 vs. −84.50 kcal/mol; Figures 3D and 3E). We hypothesized that Scramble V1 retained affinity for HIV-1 due to the low sequence diversity of the HIV-1-targeting binding domain, which is composed of 35 uracil bases out of 64 total nucleotides.

To identify *in silico* a scrambled binding domain with low predicted affinity for the HIV-1 target, we developed an automated shuffle-and-fold program to first shuffle the D4 binding domain and then fold the resultant scrambled candidate against the HIV-1 target. More than 1 × 10^5^ sequences were assessed by the program to arrive at Scramble V2, which made no or reduced contact with the HIV-1 target in representative centroid (Figure 3C; 0.00 kcal/mol) and MFE secondary structures (Figure 3F; −6.28 kcal/mol), respectively. We decided to investigate Scramble V1 and Scramble V2 in parallel to determine if HIV-1:binding domain RNA secondary structure modeled *in silico* was predictive of HIV-1 D4 targeting potential *in vitro* in HIV-1-expressing cells, and if so, if the predicted strength of interaction correlated with the extent of GCV-mediated killing by opt 2 LVVs.

**Figure 4:**
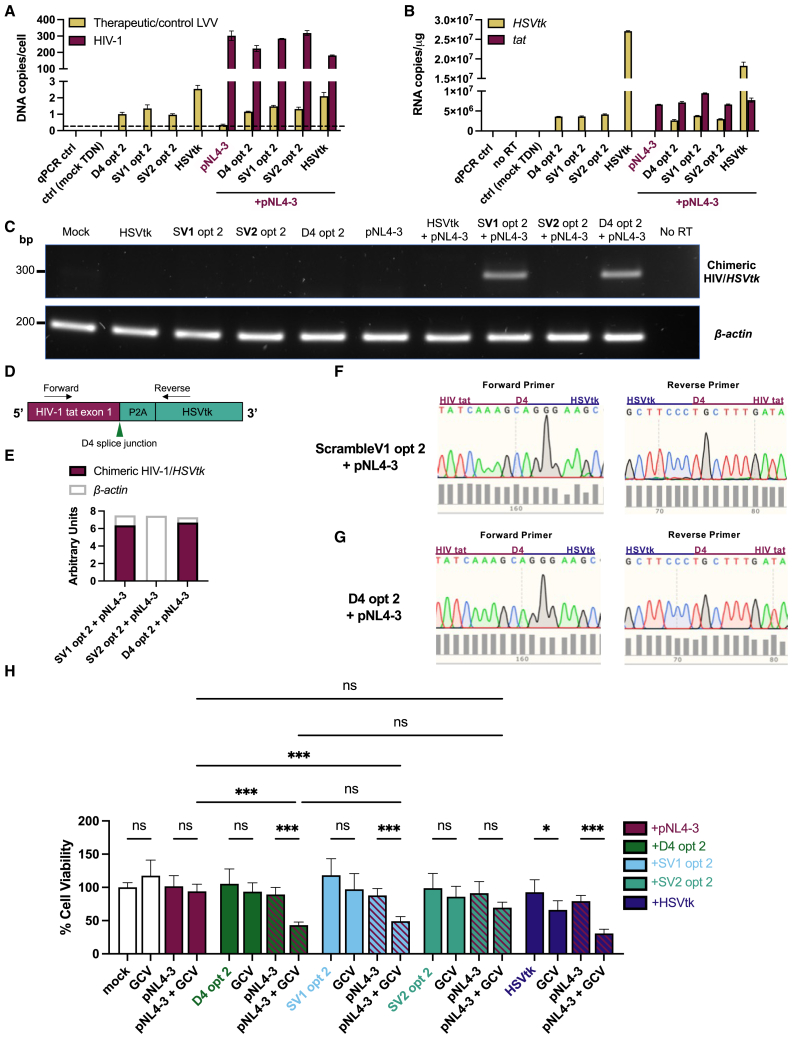
*In silico* HIV-1 RNA-targeting potential correlates with the levels of HIV-1 D4 *trans*-splicing and GCV-mediated killing achieved by opt 2 LVV in HIV-1-expressing cells (A–G) HEK293T cells were seeded in duplicate wells at 2 × 10^5^/well on day 1, transfected with 200 ng full-length HIV-1_NL4-3_ plasmid (pNL4-3) on day 2, transduced with EF1α-driven LVV panel (MOI = 2) on day 3, subjected to a media change on day 4, and lysed for DNA or RNA extraction on day 5. Mock treatments performed with media. (A) Viral DNA copies in extracted cellular DNA following delivery of opt 2/positive control LVV panel and HIV-1 pNL4-3 to HEK293T, assessed by *WPRE* and *tat* qPCRs, respectively, normalized to *ALB* qPCR. Values at the dashed line were considered background amplification but did not meet the criteria to be excluded from the analysis. (B) Levels of opt 2/positive control RNA payload and HIV-1_NL4-3_ RNA target (per microgram of total cellular RNA) in HEK293T cells, assessed by RT-qPCR for *HSVtk* and *tat*, respectively, normalized to *β-actin*. (A and B) Data presented as mean with SD (*N* = 2 qPCR replicates). (C) RT-PCR detection of putative chimeric HIV-1/*HSVtk* RNA sequences (291 bp) in HIV-1-expressing HEK293T cells following delivery of D4 opt 2 or Scramble V1 (SV1) opt 2 LVV, with *β-actin* RT-PCR (202 bp) for normalization. PCR products were gel extracted for sequencing. (D) PCR primer design for amplification of the splice junction of chimeric HIV-1/*HSVtk* transcripts (long amplicon), with the forward primer positioned in HIV-1 *tat* exon 1 and the reverse positioned in *HSVtk*. Diagram not to scale. (E) Densitometric quantification of (C). (F and G) Sanger sequencing confirmation of *trans*-spliced HIV-1/*HSVtk* RNA in HIV-1-expressing HEK293T cells following delivery of (F) Scramble V1 opt 2 or (G) D4 opt 2 LVV. Sequencing performed with *trans*-splice PCR primers. Chromatogram snapshots depict the HIV-1/*HSVtk* splice junction. (H) Viability screen in HIV-1-expressing and uninfected cells. 2 × 10^4^ HEK293T cells/well were seeded on day 1, transfected with 100 ng HIV-1 pNL4-3 on day 2, transduced with EF1α-driven LVV panel (MOI = 3) on day 3, treated with 50 μM GCV doses on days 4 and 5, and subjected to MTT cell viability assay on day 8. Mock treatments performed with media only. Data presented as mean with SD (*N* = 3 independent experiments, each performed in triplicate). ∗*p* < 0.05, ∗∗∗*p* < 0.001; one-way ANOVA with Tukey’s multiple comparisons test.

We first confirmed that transduction of EF1α-directed D4 opt 2, Scramble V1 opt 2, and Scramble V2 opt 2 LVV was comparable, with a narrow range in VCN from 1.16 to 1.48 in HIV-1_NL4-3_-expressing HEK293T determined by qPCR (Figure 4A). Produced and titrated separately, the HSVtk positive control LVV was not used for direct comparison against the opt 2 panel. We also assessed the consistency of HIV-1_NL4-3_ delivery in opt 2-transduced HEK293T cells, determining a range from 224 to 318 HIV-1 DNA copies/cell based on qPCR (Figure 4A). We hypothesize that the high number of DNA copies was due to the high efficiency of transient transfection, necessary for expression of the full-length HIV-1 molecular clone in HEK293T. Congruent with our analysis of viral DNA, opt 2 RNA payload and HIV-1 target RNA levels were broadly similar across transduced cells based on RT-qPCR for *HSVtk* and HIV-1 *tat*, respectively (Figure 4B). Taken together, the qPCR assays confirmed that the major difference between HIV-1-expressing HEK293T cells transduced with Scramble V1, Scramble V2, or D4 opt 2 would be the sequence of the binding domain in the opt 2 payload, as delivery of and expression from HIV-1 and the opt 2 LVV were comparable across the populations.

**Figure 5:**
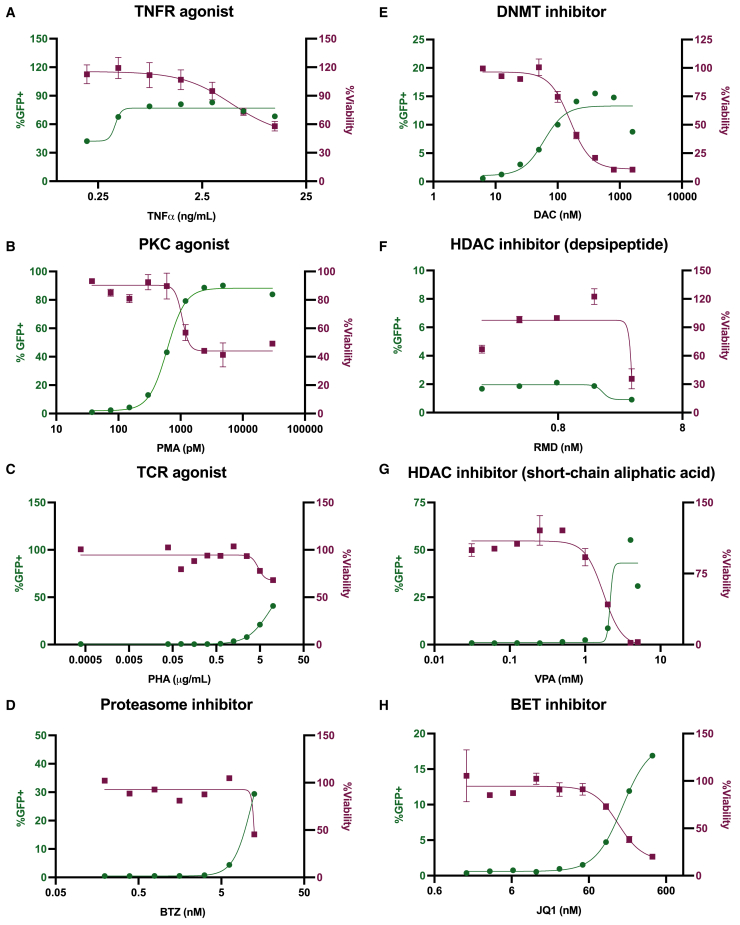
Dose-response of chronically HIV-1-infected J-Lat 10.6 cells to LRA treatment Dose-response curves for HIV-1 reactivation (green line), based on the percentage of live J-Lat 10.6 cells determined by flow cytometry to express EGFP from the HIV-R7/E^–^/GFP 5′ long terminal repeat (LTR). Dose-response curves for cell viability (red line) were based on the MTT assay. J-Lat 10.6 cells were treated with (A) the tumor necrosis factor receptor (TNFR) agonist TNFα, (B) the protein kinase C (PKC) agonist phorbol 12-myristate 13-acetate (PMA), (C) the T cell receptor (TCR) agonist phytohemagglutinin (PHA), (D) the 26S proteasome inhibitor bortezomib (BTZ), (E) the DNA methyltransferase (DNMT) inhibitor decitabine (DAC), (F) the histone deacetylase (HDAC) inhibitor romidepsin (RMD), (G) the HDAC inhibitor valproic acid (VPA), or (H) the bromodomain and extraterminal domain protein (BET) inhibitor JQ1 at the specified doses and were assayed for HIV-1 reactivation and cell viability levels subsequent to (A, C, E, G, and H) five or (B, D, and F) six additional days of incubation. Where duplicate wells were assayed, data presented as mean with SD. Across the LRA panel, relative EC_50_ values for HIV-1 reactivation were estimated to be (A) 0.40 ng/mL, (B) 607 pM*,* (C) 8.30 μg/mL, (D) 11.7 nM, (E) 60.2 nM, (G) 2.15 mM, and (H) 163 nM. The relative EC_50_ value for (F) could not be reliably determined due to low HIV-1 reactivation coupled with sharp increase in toxicity over RMD dosing range. The baseline level of GFP+ across the LRA panel ranged from 0.36% to 2.21% of unstimulated J-Lat 10.6 cells.

**Figure 6:**
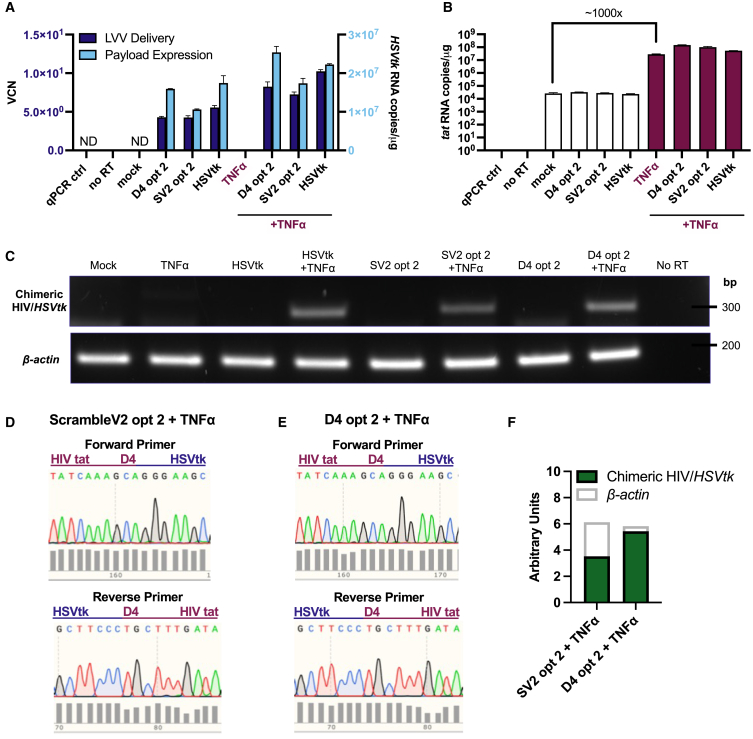
Chronically HIV-1-infected J-Lat 10.6 cells transduced with *Trans*-splicing Opt 2 LVVs can produce chimeric HIV-1/*HSVtk* mRNA when LRA treatment is used to stimulate HIV-1 expression 1 × 10^5^ J-Lat 10.6 cells were seeded in duplicate wells on day 1, stimulated with the LRA TNFα (1.56 ng/mL) on day 2, transduced with EF1α-directed LVVs at an MOI of 14 on day 3, and lysed for DNA or RNA extraction on day 5. Mock treatments performed with media. (A) Evaluation of opt 2/positive control LVV delivery and payload expression in J-Lat 10.6 cells ± TNFα stimulation. (Left axis) LVV delivery (based on VCN) assessed by *WPRE* qPCR on cellular DNA, normalized to *ALB*. (Right axis) LVV payload expression (*HSVtk* RNA copies per microgram of total cellular RNA) assessed by RT-qPCR on cellular RNA, normalized to *β-actin*. ND, below limit of detection. (B) Evaluation of HIV-1 transcription in J-Lat 10.6 cells ± TNFα stimulation. HIV-1 target expression (*tat* RNA copies per microgram of total cellular RNA) assessed by RT-qPCR on cellular RNA, normalized to *β-actin*. (A and B) Data presented as mean with SD (*N* = 2 or 3 qPCR replicates/condition). (C-F) Evaluation of HIV-1 *trans*-splicing in J-Lat 10.6 cells transduced with LVV panel ± TNFα stimulation. (C) (Top) Chimeric HIV-1/*HSVtk* splice junctions amplified by RT-PCR on cellular RNA; 291-bp amplicon expected (see Figure 4D for primer design). PCR products were gel extracted for sequencing; refer to Figure S11 for analysis of product from TNFα-stimulated cells transduced with HSVtk LVV. (Bottom) *β-actin* RT-PCR for normalization; 202-bp amplicon expected. (D and E) Chromatograms of HIV-1/*HSVtk* splice junctions from TNFα-stimulated J-Lat 10.6 cells transduced with (D) Scramble V2 opt 2 and (E) D4 opt 2 *trans*-splicing LVVs. Sequencing performed with *trans*-splice PCR primers. (F) Densitometric quantification of select lanes in (C). SV2, Scramble V2.

We next evaluated the HIV-1 D4-targeting potential of the binding domains, using RT-PCR to detect chimeric HIV-1/*HSVtk* mRNA *trans*-spliced at D4 in *in vitro* cell-based assays (Figures 4C and S8A). An alternative primer set (Figure 4D) allowed for better separation between on-target (291 bp) and nonspecific PCR products (Figure S8). Sanger sequencing confirmed the presence of *trans*-spliced products in the RNA of HIV-1_NL4-3_-expressing cells transduced with D4 or Scramble V1 opt 2 LVV (Figures 4F and 4G), consistent with the stable duplexes predicted to form between HIV-1 and binding domain RNAs (Figure 3A, 3B, 3D, and 3E). In contrast, HIV-1/*HSVtk* chimeras were not detectable in the RNA of HIV-1_NL4-3_-expressing cells transduced with Scramble V2 opt 2 LVV (Figure 4C), despite equivalent levels of *β-actin* amplification (Figure 4E). In an alternative T cell line model of HIV-1 infection, where the levels of Scramble V2 opt 2 RNA and HIV-1 target RNA (induced by LRAs; see Figure 5 for panel) were six times and 15- times higher (Figures 6A and 6B), respectively, we found that HIV-1/*HSVtk* mRNA *trans*-spliced at D4 could be detected (Figures 6C and 6D). This finding suggested that Scramble V2 is capable of weakly targeting HIV-1 D4 as per the HIV-1:binding domain MFE structure modeled *in silico* (Figure 3F), with high levels of opt 2 and HIV-1 pre-mRNA required for *trans*-spliced products to be discerned. Taken together, our data suggest that the relative stability of the HIV-1:binding domain RNA duplex predicted *in silico* correlates with the levels of *trans*-splicing at HIV-1 D4 in HIV-1-expressing cells transduced with opt 2 LVV.

We next assessed if the differences we had observed in HIV-1 *trans*-splicing potential across our opt 2 LVV panel (Figures 4C and 4E) would affect their capacity for HIV-1-expressing cell elimination with GCV. Killing potential was evaluated in HIV-1_NL4-3_-expressing and wild-type HEK293T cells (Figure 4H) to follow on from the DNA- and RNA-based assays of opt 2 LVV delivery, payload expression, and HIV-1 *trans*-splicing (Figures 4A–4G). In the absence of HIV-1, our opt 2 LVV panel was inactive as no significant GCV-mediated cytotoxic effects were induced (Figure 4H). There was, however, a highly significant difference in viability when HIV-1_NL4-3_-expressing HEK293T transduced with either D4 or Scramble V1 opt 2 were additionally treated with GCV, with a reduction from 89.32% to 43.25% associated with D4 opt 2 and a reduction from 87.93% to 49.04% associated with Scramble V1 opt 2 (Figure 4H). The viability of HIV-1-expressing cells treated with GCV alone also was significantly higher than that of cells subjected to GCV and D4/Scramble V1 opt 2 (Figure 4H), indicating that both components of the CSS were necessary for targeted cell killing. The selective and significant levels of GCV-mediated HIV-1-expressing cell elimination achieved by these LVVs (Figure 4H) dovetailed with our *in silico* and *in vitro* assays, which demonstrated that D4 opt 2 and Scramble V1 opt 2 had high HIV-1 RNA-targeting potential (Figures 3A, 3B, 3D, and 3E) and could readily induce HIV-1 RNA *trans-*splicing for completion of their AUG1-deficient *HSVtk* messages (Figures 4C and 4E–4G).

When HIV-1-expressing cells were transduced with Scramble V2 opt 2, we observed a modest decrease in viability following GCV treatment, from 91.24% to 69.51%, that was not statistically significant (Figure 4H). The viability score of HIV-1-expressing cells subjected to both Scramble V2 opt 2 and GCV was notably higher than that of HIV-1-expressing cells subjected to both D4 opt 2 and GCV; however, there was no significant difference between the two (Figure 4H). There thus was a possibility that Scramble V2 opt 2 induced a minor degree of HIV-1-dependent GCV-mediated cytotoxicity, as *in silico* (Figure 3F) and *in vitro* assays (Figures 6C and 6D) suggested that its HIV-1 RNA *trans*-splicing potential, though attenuated, was not completely abolished.

As our opt 2 LVV panel differed solely in the sequence and affinity of the binding domains for HIV-1, the results of our cell viability (Figure 4H) and *trans*-splicing assays (Figures 4C–4G) conducted in HIV-1_NL4-3_-expressing HEK293T would suggest that the extent of killing was driven by the extent of interaction between HIV-1 and opt 2 pre-mRNA molecules. This would suggest that the reduction in HIV-1-expressing cell viability by D4 opt 2 and GCV is an on-target effect driven by HIV-1 RNA *trans*-splicing at D4 (Figure 1A), potentiated by the interaction between the D4 binding domain in therapeutic pre-mRNA and the *vpu* target in HIV-1 pre-mRNA downstream of the splice donor (Figure 1B).

### The optimized D4 opt 2 LVV can hijack HIV-1 RNA splicing in chronically infected T cells induced to express HIV-1

Having demonstrated that D4 opt 2 and GCV can kill cells actively expressing HIV-1, we next investigated if our HIV-1 RNA-targeted CSS had potential to eliminate cells chronically infected with HIV-1 through shock and kill. In this proof-of-concept study, we focused on the monoclonal J-Lat 10.6 T cell line model of HIV-1 latency. The J-Lat 10.6 genome contains one full-length HIV-1 reporter provirus (HIV-R7/E^–^/GFP, based on the R7 HXB2 derivative) with the *nef* open reading frame (ORF) replaced by *EGFP* and *env* rendered non-functional by a frameshift mutation; it is thus incapable of productive infection.^42^^,^^43^^,^^44^^,^^45^ J-Lat 10.6 cells actively expressing HIV-1 were quantified through the detection of intracellular EGFP by flow cytometry using the gating strategy exemplified in Figure S9. Under basal conditions, ≤2.21% of J-Lat 10.6 cells in culture were GFP+ (data not shown), in line with previous reports that the HIV-1 provirus therein is largely quiescent.^42^

To stimulate HIV-1 transcription, we explored a panel of LRAs covering seven mechanistic classes (Figures 5 and S10), assessing HIV-1 reactivation potential and toxic side effects 5 or 6 days post treatment to align with shock-and-kill assays (section “HIV-1 reactivation assay”). Tumor necrosis factor (TNF) α, which induces the positive transcription factor nuclear factor κB (NF-κB) through TNF receptor 1/2 signaling,^46^ resulted in the maximum tolerable (MTT viability score ≥90%) level of HIV-1 reactivation (Figure 5A). At 1.56 ng/mL, ∼80% of J-Lat 10.6 cells were GFP+ based on flow cytometry (Figure 5A).

We treated J-Lat 10.6 cells with 1.56 ng/mL TNFα or media (unstimulated control) in a pilot experiment to investigate how stimulation of HIV-1 transcription with an LRA could affect our HIV-1 RNA-targeted CSS. LVV delivery and payload expression in J-Lat 10.6 cells was validated at the concentration used for shock-and-kill assays (MOI of 14) by (RT)-qPCR (Figure 6A). Treatment with TNFα a day prior appeared to have a positive effect on LVV transduction (Figure 6A). TNFα is known to upregulate the low-density lipoprotein (LDL) receptor—used by VSV-G-pseudotyped LVVs for entry^47^—in hepatocytes^48^ and endothelial cells,^49^ and we would hypothesize that lymphocytes may be similarly affected.

We next studied how TNFα affected the HIV-1 provirus within J-Lat 10.6 cells compared to those left unstimulated (Figure 6B). In support of the low-level stochastic reactivation (GFP+) we had observed by flow cytometry and fluorescence microscopy (see leftmost panel in Figure S10F), processive HIV-1 transcription could be detected in unstimulated J-Lat 10.6 cells based on HIV-1 *tat* RT-qPCR (Figure 6B). However, the levels of target HIV-1 RNA in the unstimulated population proved insufficient for discernible therapeutic *trans*-splicing with D4 opt 2, based on RT-PCR for HIV-1*/HSVtk* chimera (Figure 6C). Unstimulated J-Lat 10.6 cells thus were established as a control population in which to evaluate the effects of our HIV-1 RNA-targeted CSS in the absence of detectable HIV-1*/HSVtk* mRNA encoding nflHSVtk enzyme.

Compared to unstimulated cells, TNFα-treated J-Lat 10.6 expressed at least 1,000 times more *tat* RNA transcripts (Figure 6B), in line with high levels of HIV-1 latency reversal observed by flow cytometry (Figure 5A). We sought therapeutic HIV-1 *trans*-splicing in TNFα-stimulated J-Lat 10.6 cells transduced with D4 opt 2 LVV and observed a putative on-target signal (291 bp) by RT-PCR (Figure 6C), which was confirmed by Sanger sequencing (Figure 6E). This demonstrates that HIV-1 alternative splicing can be hijacked to functionalize a therapeutic payload in chronically infected T cells.

We included the Scramble V2 opt 2 LVV in our pilot experiment to assess if a binding domain with lower predicted affinity for HIV-1 RNA (Figures 3C and 3F) would have reduced HIV-1 *trans*-splicing potential in chronically HIV-1-infected cells compared to D4 opt 2 (Figures 3A and 3D). We found by RT-PCR (Figure 6C) and Sanger sequencing (Figure 6D) that Scramble V2 opt 2 was capable of inducing HIV-1 *trans-*splicing at the D4 donor site in TNFα-stimulated J-Lat 10.6 cells, albeit at a lower level than that observed for D4 opt 2 (Figure 6F). The functional consequences of low-affinity HIV-1:Scramble V2 interactions were more apparent in LRA-stimulated J-Lat 10.6 cells compared with our HEK293T model of HIV-1 expression, where opt 2 LVV payload and target HIV-1 RNA levels were lower (Figure 4B).

We considered HIV-1 D4 to be a promising target for therapeutic manipulation as previous reports have shown that the donor site is liable to undergo aberrant RNA splicing reactions with non-HIV-1 acceptor sites.^50^^,^^51^ Chimeric HIV-1/cellular mRNAs formed from splicing at HIV-1 D4 have been detected in primary CD4^+^ T cell models of productive infection^51^ and HIV-1 latency.^52^ In the present study, we detected an additional chimeric mRNA species formed through splicing at D4 (Figure 6C), which we found by Sanger sequencing to be composed of sequences from HIV-1 and the HSVtk positive control vector (Figure S11). The translational initiation codons gained through *trans*-splicing were not in frame with HSVtk (Figure S11). Based on BLAST analysis, the EF1α-directed full-length *HSVtk* payload shared no sequence similarity with the J-Lat 10.6 HIV-1 provirus (GenBank: MN989412.1) in lieu of a binding domain to potentiate an RNA *trans*-splicing reaction. As the chimeric mRNA species was not detected in HIV-1-expressing HEK293T cells transduced with the same LVV (Figure 4C), we hypothesize that the high levels of *HSVtk* and HIV-1 RNA in LRA-treated J-Lat 10.6 cells (Figures 6A and 6B) were a contributing factor.

### When stimulated with LRAs, chronically HIV-1-infected T cells can be killed by the optimized D4 opt 2 LVV and GCV

**Figure 7:**
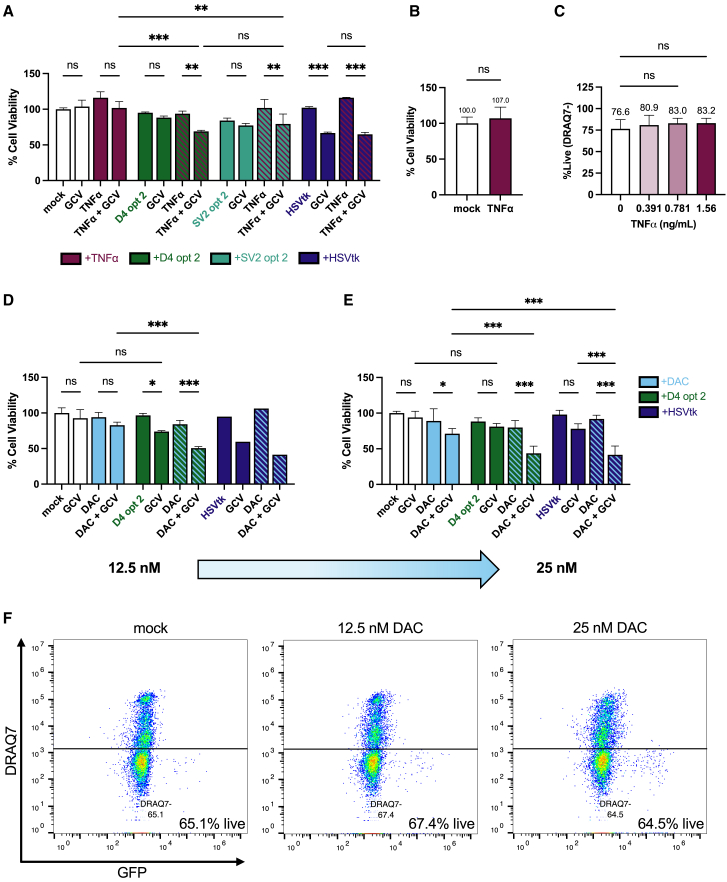
LRA treatment can influence the susceptibility of chronically HIV-1-infected J-Lat 10.6 cells to killing by an HIV-1 RNA-targeted CSS, D4 opt 2 LVV and GCV (A–C) Effect of TNFα on J-Lat 10.6 cells in isolation and in combination with HIV-1 RNA-targeted CSS for shock and kill. (A and B) Viability screen based on population-level metabolism of MTT. 5 × 10^3^ J-Lat 10.6 cells/well were seeded on day 1, treated with 1.56 ng/mL TNFα (or mock; media) on (A) day 2 or (B) day 3, transduced with EF1α-driven LVV panel (MOI = 14 or mock; media) on (A) day 3 or (B) day 2, treated with 50 μM GCV doses (or mock; media) on days 4 and 5, and assayed on day 8. (A) *N* = 3 wells/condition. SV2, Scramble V2. (B) *N* = 7 independent experiments; two or three wells/condition in each experiment. (C) Percentage of J-Lat 10.6 cell population determined to be live (DRAQ7−) by flow cytometry. *N* = 2 or 3 independent experiments, including data from experiment described in Figure 5A. Refer to Figure 5A for experimental details. (D–F) Effect of DAC on J-Lat 10.6 cells in isolation and in combination with HIV-1 RNA-targeted CSS for shock and kill. (D and E) Viability screen based on population-level metabolism of MTT. 5 × 10^3^ J-Lat 10.6 cells/well were seeded on day 1, treated with (D) 12.5 nM or (E) 25 nM DAC (or mock; media) on day 2, transduced with EF1α-driven LVV panel (MOI = 14 or mock; media) on day 3, treated with 50 μM GCV doses (or mock; media) on days 4 and 5, and assayed on day 8. (D) *N* = 3 wells/condition with exception of HSVtk TDNs (one well/condition). (E) *N* = 4–6 wells/condition across two independent experiments. (F) Flow plots depicting percentage of J-Lat 10.6 cell population determined to be live (DRAQ7−) by flow cytometry. Data from experiment described in Figure 5E; refer to Figure 5E for experimental details. (A–E) Data presented as mean with SD. (A, C, D, and E) One-way ANOVA with Tukey’s multiple comparisons test. (B) Mann-Whitney test. ns, not significant; ∗*p* < 0.05, ∗∗*p* < 0.01, ∗∗∗*p* < 0.001.

Having confirmed that *HSVtk*_ΔAUG1_ could be completed through RNA *trans*-splicing at HIV-1 D4 in chronically HIV-1-infected cells with LRA treatment, we sought to understand how the addition of GCV would affect cell viability. In our pilot experiment with TNFα, we found that the MTT viability score of J-Lat 10.6 cells transduced with D4 opt 2 was significantly reduced from 93.8% to 68.9% with GCV, while the score of those transduced with Scramble V2 opt 2 was significantly reduced from 102% to 79.3% with GCV (Figure 7A). Although not a significant difference, the more pronounced effect on viability from D4 opt 2 compared to Scramble V2 opt 2 (Figure 7A) aligned with HIV-1 RNA *trans*-splicing levels (Figure 6F). Unstimulated J-Lat 10.6 cells transduced with D4 opt 2 and Scramble V2 opt 2 LVVs did not produce detectable HIV-1*/HSVtk* mRNA (Figure 6C) and were found to be insensitive to GCV (Figure 7A). Furthermore, treatment of J-Lat 10.6 cells with TNFα and GCV alone was well tolerated (Figure 7A). Significant GCV-mediated cytotoxicity from D4 opt 2 and Scramble V2 opt 2 was thus concomitant with the detection of chimeric HIV-1*/HSVtk* mRNA in *trans*-splicing assays (Figures 7A and 6C–6F), suggesting that CSS activation was dependent on completion of *HSVtk*_ΔAUG1_ by HIV-1 RNA *trans*-splicing in chronically infected cells. Having established the connection between a specific RNA *trans*-splicing reaction (Figures 6C–6F) and GCV-mediated cytotoxicity (Figure 7A) for two LVVs, we focused on D4 opt 2 exclusively in all subsequent experiments as this therapeutic candidate was designed to have the highest affinity for HIV-1 RNA.

Through this study, we demonstrated that LRA-induced HIV-1 reactivation in chronically infected cells could be leveraged by our HIV-1 RNA-targeted CSS, though the reduction in cell viability achieved was modest (Figure 7A). We wondered if our choice of LRA could be optimized, as TNFα-induced transcription factors NF-κB and AP-1 are known to upregulate cellular anti-apoptotic and pro-proliferative genes^46^ in addition to the HIV-1 provirus.^53^ In our titration, TNFα appeared to have a positive effect on cell viability at doses ranging from 0.195 to 1.56 ng/mL, based on the MTT assay (Figure 5A).

We investigated the effect of TNFα on cell viability in seven further independent experiments at 1.56 ng/mL, the dose used for shock and kill (Figure 7B). Here, we found that the average viability score of TNFα-treated cells was higher than that of cells treated with media only (107% versus 100%), though the difference was not statistically significant (Figure 7B). As the MTT assay measures viability at the population level, scores >100% could be due to enhanced proliferation and/or enhanced survival. For better understanding, we performed live/dead staining with DRAQ7 and found that a higher percentage of cells was considered live (DRAQ7−) following treatment with TNFα (0.391, 0.781, or 1.56 ng/mL) in lieu of media, with the greatest difference observed at 1.56 ng/mL (83.2% live versus 76.6% live for media control) (Figure 7C). This effect was dose dependent as higher concentrations of TNFα (≥ 3.13 ng/mL) proved increasingly toxic in both MTT (Figure 5A) and live/dead assays (data not shown). Collectively, these results suggest that TNFα—though an attractive *in vitro* agent for the reversal of HIV-1 latency—may not be the most conducive to J-Lat 10.6 cell elimination.

Of our panel (Figure 5), we found that the DNA methyltransferase inhibitor (DNMTi) decitabine (DAC) demonstrated the most promise for shock and kill in combination with our HIV-1 RNA-targeted CSS, D4 opt 2 and GCV (Figures 7D and 7E). At 12.5 nM DAC, the viability of J-Lat 10.6 cells transduced with D4 opt 2 was significantly reduced from 84.0% to 50.6% with GCV (Figure 7D); at 25 nM DAC, the viability of D4 opt 2-transduced cells was reduced further still, from 79.8% to 43.6% in the presence of GCV (Figure 7E). The viability score of DAC-treated cells subjected to both D4 opt 2 and GCV also was significantly lower than that of DAC-treated cells subjected to GCV alone (Figures 7D and 7E). In contrast, there was no statistically significant difference in the viability scores of unstimulated cells comparing those subjected to both D4 opt 2 and GCV to those subjected to GCV alone (Figures 7D and 7E). Collectively, these observations suggested that DAC treatment made chronically HIV-1-infected cells susceptible to our HIV-1 RNA-targeted CSS.

Although TNFα was the superior LRA for HIV-1 reactivation in our flow cytometry assays (Figures 5A and 5E), treatment with DAC ultimately enabled higher reductions in cell viability with D4 opt 2 and GCV. From the MTT viability scores, we calculated that 45.4% of cells stimulated with DAC (25 nM) and transduced with D4 opt 2 were effectively eliminated with GCV (Figure 7E), compared to 26.6% when TNFα (1.56 ng/mL) was used as the LRA (Figure 7A). We hypothesize that the extent of killing by our HIV-1 RNA-targeted CSS was influenced by the effect of LRA treatment on the chronically HIV-1-infected cells themselves. At the doses used for shock and kill (12.5 or 25 nM), DAC appeared to have a neutral effect on J-Lat 10.6 cell survival based on DRAQ7 staining (Figure 7F), in contrast to the positive effect observed from TNFα (Figure 7C). Intriguingly, we also observed that DAC (Figure 7E), unlike TNFα (Figure 7A), could significantly increase the sensitivity of J-Lat 10.6 cells to the full-length CSS used as a positive control. Based on the viability scores from our MTT assays, 54.8% of cells stimulated with DAC (25 nM) and transduced with HSVtk were effectively eliminated with GCV, compared to 20.4% when cells were left unstimulated (Figure 7E). We wondered if this effect was due to an enhancement in LVV delivery and/or payload expression by DAC. However, in a preliminary *HSVtk* RT-qPCR, we found that LVV positive control payload levels were comparable in unstimulated J-Lat 10.6 cells transduced with HSVtk compared to those first treated with DAC at 25 nM (Figure S12A).

We considered the mechanism of action of DAC. Following phosphorylation by cellular kinases, DAC is incorporated into DNA as DAC-TP, which forms an irreversible covalent bond with DNMTs to inhibit enzymatic activity and promote global demethylation.^54^ Methylated CpG islands in the HIV-1 provirus are known to enforce latency as platforms for transcriptional repressors such as methyl-CpG binding domain protein 2.^55^^,^^56^ In J-Lat 10.6 cells, DAC-induced HIV-1 reactivation was confirmed at both the protein (Figure 5E) and RNA level (Figure S12B) by flow cytometry and RT-qPCR, respectively. In the clinic, DNA hypomethylation can be induced with well-tolerated doses of DAC; however, at higher doses, adducts of DAC-TP/DNMT become excessive and cellular DNA synthesis can instead be inhibited.^54^ Due to their complementary modes of action, we hypothesize that DAC-TP synergizes with GCV-TP, the end-product of HSVtk activity, to increase the cytotoxicity of GCV-TP-mediated DNA damage. Synergy between DAC-TP and GCV-TP was previously observed in the context of equid herpesvirus-1 (EHV-1) inhibition and was linked to the specific incorporation of both GCV-TP and DAC-TP into EHV-1 DNA.^57^ Based on these analyses, we posit that DAC could be an attractive component of an LRA regimen for shock and kill with our HSVtk/GCV-based, HIV-1-dependent CSS and would merit a follow-up investigation to further explore its mechanism of action.

A treatment that could reduce the size of the HIV-1 reservoir remains elusive. Having demonstrated that our optimized HIV-1 RNA-targeted CSS has the potential to kill cell lines actively expressing HIV-1 and those chronically infected when combined with LRAs, further study and refinement of our shock-and-kill strategy in primary cell models of HIV-1 infection would be warranted.

## Discussion

The objective of this study was to advance the therapeutic potential of an HIV-1-targeted HSVtk/GCV CSS that hijacks HIV-1 RNA splicing—a process essential for viral replication^14^—to selectively eliminate HIV-1-infected cells. HIV-1 D4, our target splice donor, is required for the biogenesis of completely spliced HIV-1 mRNAs encoding proteins critical for productive replication (Tat, Rev)^58^ and pathogenesis (Nef).^59^ As the default behavior of the cellular spliceosome is to remove introns from unspliced pre-mRNA transcripts, some completely spliced transcripts are produced whenever transcription from the HIV-1 provirus is processive,^50^^,^^60^ with concomitant opportunity for therapeutic manipulation at D4. Negative feedback from Rev keeps the level of completely spliced transcripts in check by diminishing the pool of unspliced precursor RNAs in the nucleus.^61^ Although these dynamics would likely modulate the level of *trans*-spliced HIV-1 *tat/HSVtk* mRNA from which nflHSVtk is translated, once expressed, the cell suicide enzyme can be highly stable in mammalian cells (half-life of ∼35 h),^62^ and GCV-TP, the cytotoxic end-product of HSVtk activity, is known to have good intracellular persistence^62^^,^^63^ (half-life of ∼12–18 h^63^).

Given the chronic nature of HIV-1 disease, where some healthy cells will eventually become infected and other latently HIV-1-infected cells will eventually reactivate,^3^ long-term expression of anti-HIV-1 gene therapy is desirable. With an LVV-based delivery system, autologous CD4^+^ T cells derived from leukapheresis collections could be transduced *ex vivo* for stable expression of the HIV-1-targeted cell suicide gene, followed by reinfusion of the edited cells into HIV-1-infected patients and treatment with GCV.

We developed our HIV-1-targeted CSS^16^ into an LVV-based gene therapy and confirmed that the approach could be used to functionalize AUG1-deficient *HSVtk* by HIV-1 RNA *trans*-splicing in HIV-1-expressing CD4^+^ lymphoid cells; however, alternative AUGs in the *HSVtk*_ΔAUG1_ sequence were a liability to the selectivity of the therapeutic payload as these allowed for HIV-1-independent translation of truncated HSVtk polypeptides known to have catalytic activity.^23^ Through our lead therapeutic candidate D4 opt 2, our group has demonstrated that modification of *HSVtk* AUG46 and AUG60 can better constrain HSVtk/GCV-mediated cytotoxicity to the target cell population. This finding could benefit other therapeutic areas such as cancer, where analogous approaches have been used to activate HSVtk/GCV by RNA *trans*-splicing.^64^^,^^65^ The present study demonstrates that D4 opt 2 could induce GCV-mediated cytotoxicity in the presence of HIV-1, suggesting that nflHSVtk formed through HIV-1-targeted *trans*-splicing was catalytically active despite substitutions to amino acid residues 46 and 60 (M46L and M60I), in agreement with previously published reports on HSVtk structure and function.^23^^,^^66^ Of note, the most dramatic effect on HIV-1-expressing cell proliferation was achieved when D4 opt 2 expression was directed by the constitutive human EF1α promoter. We selected this promoter as it is known to direct high levels of transgene expression in primary T lymphocytes and CD34^+^ hematopoietic stem cells (HSCs), the ultimate potential targets of our therapeutic approach.^67^

Constitutive expression of *trans*-splicing RNA is attractive as it would theoretically allow for our HIV-1-targeted CSS to be active at the earliest stage in viral gene product synthesis; however, with progression toward the clinic, this will need to be balanced against the potential to induce off-target effects in healthy cells. The HIV-1 RNA-targeting binding domain was demonstrated previously to be the superior of a 10-candidate panel tested *in vitro* for both on- and off-target toxicity.^16^ As such, the focus of the present study was limited to on-target *trans*-splicing between therapeutic and HIV-1 pre-mRNAs, as it was essential to confirm that the modifications we made in HIV-1-targeting D4 opt 2 did not inadvertently interfere with its intended function. Here, we found that the affinity of opt 2 payloads modeled *in silico* for HIV-1 RNA correlated with their potential to induce HIV-1 *trans*-splicing and killing of HIV-1-expressing cells. Importantly, in the absence of HIV-1, significant GCV-mediated cytotoxicity was not induced in the cell lines we examined. Although beyond the scope of the present study, single-cell analyses of D4 opt 2 integration site(s) and splicing patterns could be used in future investigations to provide more understanding into potential off-target effects in primary cells and whether these are stochastic or recurring following LVV transduction. Alternative non-integrating LVVs^68^ or other gene delivery systems such as the recombinant adenoviral vector Ad5/35^69^ could be used for comparison.

We considered HIV-1-expressing Jurkat and HEK293T cell lines to be appropriate for assessing potential optimizations of our HIV-1 targeted CSS as they have previously enabled better understanding of HIV-1 infection^42^^,^^70^^,^^71^ and, most importantly, HIV-1 alternative splicing,^72^ the stage of viral replication targeted by our approach. Our ultimate model for active HIV-1 expression, HEK293T cells transfected with full-length HIV-1_NL4-3_, was used by Emery et al. to study the HIV-1 transcriptome by next-generation sequencing (NGS).^72^ Collectively, transcriptome studies have suggested that the mode of delivery of HIV-1 does not markedly alter the downstream process of HIV-1 alternative splicing in the particular cell types examined^72^^,^^73^ and that splicing patterns are similar in transduced primary CD4^+^ T cells and transfected cell lines not naturally permissive to HIV-1.^73^ Of note, the precise composition of the HIV-1 transcriptome is known to vary by viral strain^72^ and cellular donor.^60^ Although further work is needed to understand the extent to which this influences the activity of our HIV-1 RNA-targeted CSS, the HIV-1_NL4-3_ molecular clone used in our study was demonstrated by Emery et al. to be a good representative of the donor and acceptor splice site combinations utilized by a range of HIV-1 subtype B clinical isolates.^72^ Furthermore, the ability of our CSS to induce HIV-1 *trans*-splicing and killing of LRA-stimulated, chronically infected J-Lat 10.6 cells is encouraging as these cells harbor an HIV-1 molecular clone based on the R7 derivative of HXB2, an alternative subtype B strain.^42^^,^^44^^,^^45^

Chronically HIV-1 infected cells are a barrier to cure and can harbor the virus in a reversible state of latency. Although LRAs of various mechanistic classes can be used to induce HIV-1 expression, this has not significantly increased the vulnerability of HIV-1-harboring cells to viral cytopathic effects and/or cytotoxic immune cells in clinical trials,^10^^,^^12^ as originally posited by the shock-and-kill approach for HIV-1 reservoir reduction.^6^ Having demonstrated that D4 opt 2/GCV could antagonize cells actively expressing HIV-1 RNA, we hypothesized that killing of chronically infected cells may be possible when HIV-1 expression was enhanced with LRAs. Using the J-Lat 10.6 model of chronic HIV-1 infection, we found that LRA treatment could be leveraged by our HIV-1 RNA-targeted CSS to reduce cell viability, suggesting a potential approach to help enhance the kill in shock and kill.

Intriguingly, the LRAs we examined appeared to affect CD4^+^ T cells in other ways besides HIV-1 reactivation status that influenced how susceptible they were to our cytotoxic stimuli. Such a phenomenon has previously been observed for the PKC agonist class of LRAs, with prostratin and bryostatin-1 found by French et al. to help protect uninfected primary CD4^+^ T cells from apoptosis induced by the DNA damage agent etoposide.^74^ Although these LRAs exhibited good HIV-1 reactivation potential, French et al. posited that their effect on cell survival could ultimately undermine the outcome of shock and kill.^74^ In the present study, we found that TNFα, though the superior of our panel for well-tolerated HIV-1 latency reversal, was not the optimal LRA to use with our HIV-1-targeted CSS for elimination of chronically HIV-1-infected cells. Although the response of HIV-1-infected cells to TNFα is known to vary dramatically depending on the cellular context and the extent of exposure,^75^ at the dose we used for shock and kill, the cytokine appeared to positively affect the viability of chronically HIV-1-infected J-Lat 10.6 cells. TNFα has previously been implicated in the persistence of HIV-1-infected CD4^+^ T cells^76^ and monocyte-derived macrophages^77^; however, further investigation was outside the scope of the present study, which focused on identifying promising LRA candidate(s) to use in combination with D4 opt 2 and GCV.

We investigated a range of LRAs of diverse mechanistic classes and found that the DNMTi DAC appeared to be uniquely suited to our HIV-1 RNA-targeted CSS, capable of perturbing HIV-1 latency and amplifying HSVtk/GCV-mediated cell killing. As the HSVtk/GCV strategy for cell elimination has been applied to a range of targets, including cancerous tissues,^78^ study of DAC in combination would be warranted beyond the context of HIV-1 infection. In such studies, selective expression of the cell suicide enzyme, such as through targeted RNA *trans*-splicing, may be particularly important for constraining cytotoxicity to diseased cells.

As an LRA, DAC may also have the potential to work in concert with antiretroviral therapy to prevent inadvertent HIV-1 reservoir expansion following latency reversal, as prior reports suggest that the DNMTi can interfere with HIV-1 reverse transcription.^79^^,^^80^ This phenomenon was not a focus of the present investigation as J-Lat 10.6 cells do not produce infectious HIV-1 particles^81^; however, we did consider how our LVVs, which also undergo reverse transcription,^19^ could be affected. As studies have shown that DAC best antagonizes HIV-1 replication when treatment occurs at the same time as infection,^80^ we performed DAC treatments 24 h prior to LVV transduction to mitigate potential interference, and found based on the HSVtk positive control that LVV expression and activity were not deleteriously affected.

Further study of DAC in combination with our HIV-1 RNA-targeted CSS would be of merit as the DNMTi has already reached the clinic, approved by the US Food and Drug Administration (FDA) for treatment of myelodysplastic syndromes^82^ and currently under investigation (NCT05230368) in PLWH as part of an LRA cocktail. Treatment of PLWH with a cocktail of LRAs of different mechanistic classes, rather than a single LRA, has been suggested as a means to make shock and kill more effective.^10^ In the present study, we found that DAC alone enabled 45% of J-Lat 10.6 cells to be killed by our HIV-1 RNA-targeted CSS, and we would hypothesize that the inclusion of additional LRAs could enhance our approach further still as we progress toward cells from HIV-1-infected donors.

Of note, the response to reactivation stimuli of cells from PLWH has yet to be fully recapitulated by a cellular model of HIV-1 latency, based on a comprehensive study by Spina et al. of both primary cells and cell lines.^83^ To begin to understand if our approach may have potential in the latency context, we elected to study the well-established^84^ J-Lat cell line model of chronic HIV-1 infection,^42^ which exhibits a number of similarities to patient-derived lymphocytes in response to LRA treatment.^83^ Although U1 and ACH2 cell lines also were considered, latency in these models is known to be enforced by mutations in HIV-1 *tat* and transactivation response (TAR), key sequences for HIV-1 transcription,^4^^,^^81^ whereas J-Lat lines retain the wild-type copies.^83^

Using the J-Lat 10.6 model of chronic HIV-1 infection, we found with LRA treatment that D4 opt 2 could induce HIV-1 RNA *trans*-splicing and that the presence of chimeric HIV-1/*HSVtk* mRNA was associated with significant GCV-mediated reductions in viability. For better understanding of the non-selective effects that could be induced by our shock-and-kill strategy, we studied three key J-Lat populations: unstimulated cells subjected to D4 opt 2/GCV, to monitor the effects of the HIV-1 RNA-targeted CSS in the absence of discernible HIV-1 *trans*-splicing; LRA-stimulated cells subjected to D4 opt 2 alone, to assess for LVV toxicity; and LRA-stimulated cells subjected to GCV alone, to assess for drug toxicity. Having demonstrated that D4 opt 2/GCV may be a promising strategy to pair with LRAs for killing of chronically HIV-1-infected cells, a follow-up investigation would be of merit in primary cell models of HIV-1 latency,^83^ which would allow for parallel analysis of matched uninfected controls from the same donor.

Subsequent investigations of our HIV-1 RNA-targeted CSS for shock and kill should additionally consider the mechanism(s) by which chronically HIV-1-infected cells can succumb to—or resist—HSVtk/GCV-induced DNA damage^85^ following LRA treatment. The ultimate targets of our therapeutic approach, HIV-1-infected primary CD4^+^ T cells and CD34^+^ HSCs, will be essential for this study as the cell death pathways activated by HSVtk/GCV are known to vary by cell type.^86^ For instance, HSVtk/GCV activity can additionally be mediated through a bystander effect, in which GCV-TP is passed between cells in close contact—canonically but not exclusively by gap junctions—to trigger death in those that have not successfully taken up the *HSVtk* transgene.^86^^,^^87^^,^^88^ The extent of the bystander effect and mode by which it occurs may vary in the cell lines studied in this work; however, we would hypothesize based on current evidence that this phenomenon could occur in primary cells naturally susceptible to HIV-1 infection. Connexin-43 hemichannels and gap junctions have been shown to play a role in signaling at the immunological synapse—facilitating the exchange of charged molecules such as ATP^89^—which is known to be hijacked by HIV-1 for cell-to-cell spread.^90^^,^^91^^,^^92^ It remains to be investigated if transfer of charged GCV-TP molecules alongside HIV-1 could occur in such instances and if this would be sufficient to eliminate newly infected cells, which would prevent further virion production and onward spread.

A further key consideration for the continued development of our anti-HIV-1 gene therapy candidate will be the difficulty of ensuring that the HIV-1-targeted cell suicide gene is delivered to all infected cells. Toward the moonshot goal of a functional HIV-1 cure, Crooks et al. suggested that a >6-fold reduction in the inducible, replication-competent HIV-1 reservoir would be a promising and clinically significant outcome.^93^ Clinical efficacy will be shaped by transduction efficiency and may be challenged further by access to HIV-1-harboring tissues beyond the peripheral blood, which is currently limited.^18^ As the gene therapy field evolves, so too may our approach. A combination of different therapeutic modalities may be needed to effectively address HIV-1 persistence; however, Pandit and De Boer have argued that inclusion of an HIV-1-targeted CSS could be critical.^94^

HIV-1 RNA is a currently unexploited therapeutic target that is vulnerable to manipulation. In this report, we have advanced a therapeutic strategy that uses HIV-1 pre-mRNA to functionalize by *trans*-splicing an incomplete pre-mRNA message encoding a cell suicide enzyme, such that cytotoxicity is conditional on HIV-1 expression at the RNA level. Our study is a proof of concept that our lead therapeutic candidate D4 opt 2/GCV has the potential to antagonize the viability of cells actively expressing HIV-1 and those induced to express HIV-1 with LRAs. As cellular reservoirs continue to fuel HIV-1 persistence in PLWH, further study and development of our HIV-1-targeted CSS would be of merit, particularly in primary cell models of acute and latent HIV-1 infection.

## Materials and methods

### Cell culture

HEK293T (ATCC) and Jurkat/Jurkat-based T cell lines (clone E6-1, ATCC; and J-Lat 10.6, NIH AIDS Reagent Program catalog #9849) were cultured at 37°C in 5% CO_2_ in DMEM (Gibco) and RPMI (Gibco), respectively, with 10% fetal bovine serum (Gibco), 100 U/mL penicillin (Gibco), and 100 μg/mL streptomycin (Gibco). All cell lines were confirmed to be mycoplasma free by the Research Instrumentation and Cell Services core facility at Cancer Research UK (Cambridge Institute). Transfections were carried out in HEK293T cells with Trans-IT-LT1 (Mirus), according to the manufacturer’s instructions.

### Chemicals

A 15× solution of 750 U/mL Benzonase endonuclease (Merck) was prepared according to Sastry et al.^95^ with 750 mM Tris pH 8 and 15 mM MgCl_2_ for dilution in lentiviral supernatant to 1×. Thiazolyl blue tetrazolium bromide (MTT; Merck) was diluted to 5 mg/mL in PBS and sterilized through a 0.20-μm filter.

Ganciclovir (GCV; Merck) powder was resuspended in DMSO to create 195.9 mM (50 mg/mL) or 50 mM stocks. LRA stocks were prepared according to manufacturers’ recommendations as follows. Phorbol 12-myristate 13-acetate (PMA; Sigma), romidepsin (RMD; Active Motif), 5-aza-2′-deoxycytidine (DAC; Abcam), and JQ1 (Abcam) powders were dissolved in DMSO. The sodium salt of valproic acid (VPA; Cayman Chemicals) was dissolved in ethanol. Phytohemagglutinin (PHA; Sigma) and TNF-α (Sino Biological) were reconstituted in sterile water and PBS, respectively. A ready-made solution of bortezomib (BTZ) in DMSO was obtained from Stratech Scientific. Aliquots were frozen at −20°C. For treatment of cells, drug aliquots were thawed and diluted further in media. The concentration of drug in cell culture on the day of treatment is reported.

### Development of scrambled binding domain

HIV-1 D4 was targeted for *trans*-splicing through a binding domain in the therapeutic RNA payload complementary to the downstream HIV-1 *vpu* sequence in HIV-1 pNL4-3 (GenBank: AF324493.2)^16^:

5′-AGAAUAUAGGAAAAUAUUAAGACAAAGAAAAAUAGACAGGUUAAUUGAUAGACUAAUAGAAAGA-3′.

The D4-targeted binding domain (D4) was as follows:

5′-UCUUUCUAUUACUCUAUCAAUUAACCUGUCUAUUUUCCUUUGUCUUAAUAUUUUCCUAUAUUCU-3′.

The D4 binding domain was scrambled to create controls with lower affinity for HIV-1 RNA. Scrambled binding domain candidates under consideration met the following three criteria: (1) no significant similarity based on primary structure BLAST alignment (https://blast.ncbi.nlm.nih.gov/Blast.cgi) to sequences within the standard (nucleotide collection) and human genomic plus transcript databases, HIV-1 pNL4-3, and the D4 binding domain; (2) no introduction of additional splice sites to RNA payload based on Splice Site Prediction by Neural Network tool (https://www.fruitfly.org/seq_tools/splice.html; forward strand; default minimum splice site scores); and (3) no introduction of additional ORFs to RNA payload based on ORF Finder (https://www.ncbi.nlm.nih.gov/orffinder/; “ATG” and alternative initiation codons with all other search parameters set to default).

Interactions between putative scrambled binding domains and the HIV-1 target were modeled *in silico* using methodology adapted from D’Souza et al.,^40^ with a 138-nt sequence submitted to the RNAfold web server (http://rna.tbi.univie.ac.at/cgi-bin/RNAWebSuite/RNAfold.cgi) comprising the 64-nt HIV-1 target, a 10-nt spacer (forced to be single stranded), and the 64-nt scrambled binding domain.

Two methods were undertaken to scramble the D4 binding domain. In the first, D4 was split into 35- and 29-nt fragments in order to be shuffled by the GenScript Sequence Scramble tool (https://www.genscript.com/tools/create-scrambled-sequence). Shuffled fragments were then concatenated to create the binding domain, Scramble Version 1:

5′-AUCUUAUUCUUAUUGCCUACUAUCUAUUCCUUAUAAUUCUUCUUAUUAUCUAUCGUUAUUCUUC-3′.

In the second, a “shuffle-and-fold” Python script was written to shuffle the D4 binding domain and perform RNA secondary structure modeling with the HIV-1 target.

Setup: D4 binding domain, HIV-1 target, and spacer constraint were represented as standard Python strings to enable manipulation. ViennaRNA was installed and added to system PATH. Multiprocessing module was used to enable multiple instances (10 chosen) of RNAfold to be run in parallel. Each process contained the following steps:

Step 1. Shuffle D4 binding domain using “random.sample” function.

Step 2. Concatenate HIV-1 target sequence and shuffled binding domain with 10-nt spacer to create duplex modeling sequence.

Step 3. Use “multiprocess.Popen” to run RNAfold within process. Input the duplex modeling sequence into RNAfold STDIN with 10-nt spacer constrained to be single stranded.

Step 4. Decode RNAfold output and extract the calculated MFE structure and number of unpaired nt. Pass all data to the central process.

Output: Central process collates all RNAfold outputs. Duplex modeling sequences with ≥110 unpaired nucleotides (out of 138 nt total) according to MFE secondary structure model are printed along with the dot-bracket RNA secondary structure of the duplex and MFE. A visual representation was generated using Forgi.

Scramble Version 2 was selected from this process as the binding domain was predicted *in silico* to have low affinity for the HIV-1 RNA target. The sequence was

5′-UUAUACCUUUUUCCUUACUUCAUUUUUUUACCCUAAUUUUACCUUUACAUUUGACCUUUUAAGU-3′.

### Cloning

#### Construction of CkRhsp promoter

A version of the CkRhsp promoter was constructed according to Farazmandfar et al.^21^ and references therein.^96^ The chicken *β-actin* promoter sequence of the *Gallus gallus* cytoplasmic *β-actin* gene, −270 to −19 (nt 273 to nt 524; GenBank: X00182.1) with reference to the putative transcriptional start (nt 544), was fused *in silico* to a 78-nt truncated HIV-1_HXB2_ R sequence (nt 454 to nt 531; GenBank: K03455.1) that included the TAR element upstream of the *hsp* promoter sequence of the *Drosophila melanogaster* heat-shock locus 87C1:distal *hsp70* gene, −89 to +62 (nt 1,424 to nt 1,574; GenBank: AH007395.1) relative to the transcriptional start (nt 1,513). The assembled promoter sequence was flanked with the *Spe*I restriction enzyme site at the 5′ end and *Nhe*I at the 3′ end. The ATG nucleotides starting at position 99 of the assembled sequence were replaced with CTC. The assembled CkRhsp promoter was then gene synthesized by GeneArt synthesis (Thermo Fisher Scientific) and subcloned into the CMV-BD1-D4-pVAX-1 and CMV-HSVtk-pVAX-1 constructs previously produced by Ingemarsdotter et al.^16^ by digestion with *Nhe*I and *Spe*I followed by ligation to replace the cytomegalovirus (CMV) promoter.

#### Generation of *BbvCI* mutant

The *Bbv*CI sequence flanking the binding domain in the construct CkRhsp-BD1-D4-pVAX-1 was mutated to remove a potential splice acceptor site, predicted with the Splice Site Prediction by Neural Network tool, using the QuickChange XL site-directed mutagenesis kit (Agilent Technologies) according to the manufacturer’s recommendations with 10-ng plasmid template and site-directed mutagenesis primers described in Table S1.

The following mutagenic PCR conditions were used: 95°C for 1 min; 18 cycles of 95°C for 50 s, 60°C for 50 s, and 68°C for 4 min; and 68°C for 7 min. The PCR products were digested for 1 h at 37°C with *Dpn*I and transformed into XL10-Gold ultracompetent cells (Agilent Technologies). Positive clones were confirmed by sequencing. The resulting construct was known as CkRhsp-BD1-D4 opt 1-pVAX-1.

#### Generation of HSVtk mutants

The *HSVtk* translational initiation mutation at *HSVtk* ATG_46_ was generated by site-directed mutagenesis using QuickChange II XL site-directed mutagenesis kit (Agilent Technologies) according to the manufacturer’s instructions with 10-ng plasmid template and site-directed mutagenesis primers described in Table S1.

Mutagenesis of ATG_46_ was confirmed by sequencing and 10 ng of the resulting plasmid was used as the template in a second round of PCR to mutate ATG_60_ using Phusion HF DNA polymerase with 1× Phusion HF reaction buffer (New England Biolabs), 125-ng forward and reverse mutagenic primers described in Table S1, 1 μL of dNTP mix (Agilent Technologies), and 3 μL of Quicksolution (Agilent Technologies). Mutagenesis PCR thermal cycling conditions and post-processing steps were performed as previously described. The resulting optimized *HSVtk* domain was subcloned into the CkRhsp-BD1-D4 opt 1-pVAX-1 backbone by digestion with *Pst*I and *Mlu*I followed by ligation to generate CkRhsp-BD1-D4 opt 2-pVAX-1.

#### Generation of RNA *trans*-splicing lentiviral transfer plasmids

To facilitate subcloning of RNA *trans*-splicing cassettes from pVAX-1 into the third-generation lentiviral gene transfer plasmid pSico (Addgene plasmid #11578), a shuttle plasmid was created using CkRhsp-BD1-D4 opt 2-pVAX-1 as template. The *Xba*I restriction enzyme site was introduced at the 5′ end of the cassette and the *Xho*I site was introduced toward the 3′ end upstream of the poly(A) sequence through two rounds of mutagenic PCRs using the QuickChange XL site-directed mutagenesis kit (Agilent Technologies) according to the manufacturer’s instructions with 10-ng plasmid template and primers described in Table S1. Mutagenesis PCR thermal cycling conditions and post-processing steps were performed as previously described.

The remaining therapeutic *trans*-splicing domains (CkRhsp-BD1-D4, CkRhsp-BD1-D4 opt 1) and CkRhsp-HSVtk positive control were subcloned into pVAX-1 shuttle plasmids using *Bcu*I and *Pst*I. Triple digestion of the pVAX-1 shuttle backbone with *Xba*I, *Xho*I, and *Nsb*I facilitated isolation of the RNA *trans*-splicing or positive control cassette between *Xba*I and *Xho*I. Cassettes were ligated into the pSico lentiviral backbone with *Xba*I and *Xho*I. The constructs were known as CkRhsp-D4, CkRhsp-D4 opt 1, CkRhsp-D4 opt 2, and CkRhsp-HSVtk.

#### Replacement of CkRhsp with EF1α promoter

In next-generation *trans*-splicing cassettes, CkRhsp was replaced with the EF1α promoter. To facilitate subcloning steps, the *Xba*I and *Bcu*I restriction sites were introduced to the 5′ end of the EF1α promoter in pEF-GFP (Addgene plasmid #11154) and *Nhe*I was introduced to the 3′end by mutagenic PCR with 100-pg plasmid template, 1.25 U of GoTaq DNA Polymerase (Promega), 1× GoTaq Buffer (Promega), 200 μM PCR nucleotide mix (Promega), nuclease-free water (Promega), and 200 nM forward and reverse primers described in Table S1. Thermal cycling conditions were as follows: 95°C for 2 min; 30 cycles of 95°C for 30 s, 60°C for 30 s, and 72°C for 1 min 11 s; 72°C for 5 min; and 4°C indefinite hold. The amplicon was subcloned into a TOPO plasmid using the TOPO TA Cloning Kit (Invitrogen) according to the manufacturer’s recommendations to culminate in EF1α-TOPO.

The EF1α promoter was subcloned from EF1α-TOPO into CkRhsp-D4 opt 2 and CkRhsp-HSVtk by digestion with *Xba*I and *Nhe*I followed by ligation, replacing the CkRhsp promoter. The ligations were transformed into Top 10 F′ cells and positive clones were confirmed by sequencing. The constructs were known as EF1α-D4 opt 2 and EF1α-HSVtk.

#### Replacement of D4 with scrambled binding domain

Scramble Version 1 and 2 (V1 and V2) binding domains were synthesized through GeneArt gene synthesis (Thermo Fisher Scientific). The restriction sites necessary for replacement of the binding domain within the *trans*-splicing cassette also were present in the pSico backbone, necessitating a multi-step subcloning process. Scramble V1 and V2 binding domains first were subcloned into CkRhsp-D4 opt 2 in the pVAX-1 shuttle by digestion with *Nhe*I and *Mlu*I, replacing the D4 binding domain. Triple digestion of the pVAX-1 backbone with *Xba*I, *Xho*I, and *Nco*I facilitated isolation of the CkRhsp-driven Scramble opt 2 *trans*-splicing cassette between *Xba*I and *Xho*I. Cassettes were ligated into the pSico backbone with *Xba*I and *Xho*I. The CkRhsp promoter was then replaced with the EF1α promoter by digestion with *Xba*I and *Nhe*I as previously described. Intermediate and final EF1α-Scramble V1 opt 2 and EF1α-Scramble V2 opt 2 constructs were verified by sequencing.

### Preparation of therapeutic/control LVV and non-replicating HIV-1: Optimized method

An optimized method for lentivirus preparation was developed with reference to Dull et al.,^19^ Tiscornia et al.,^97^ Kutner et al.,^98^ Cribbs et al.,^99^ and Sastry et al.^95^ For each preparation, 5 × 10^6^ HEK293T cells were plated in triplicate in 10-cm-diameter dishes in the evening on day 1. To produce the VSV-G-pseudotyped non-replicating HIV-1 vector, cells were transfected 24 h later on day 2 with 10 μg of pNL4-3ΔE-EGFP (NIH AIDS Reagent Program #11100) and 3.5 μg pCMV-VSV-G (Addgene plasmid #8454). For VSV-G-pseudotyped therapeutic and control LVV, 10 μg of lentiviral transfer plasmid and 3.5 μg of pCMV-VSV-G were transfected along with 6.5 μg of pMDLg/pRRE (Addgene plasmid #12251) and 2.5 μg of pRSV-Rev (Addgene plasmid #12253) (Figure S3A). Media was changed the morning of day 3. The morning of day 4, LVV supernatants (∼10 mL) from the three plates were combined and stored overnight at 4°C, and the media was replenished. The morning of day 5, LVV supernatant was harvested a second time and, alongside the first harvest, was clarified from cellular debris (2,103 × *g*, 10 min), incubated with 50 U/mL Benzonase (30 min at 37°C) for removal of LVV plasmids, and passed through 0.45-μm surfactant-free cellulose acetate syringe filters (Sartorius). The ∼30 mL of LVV supernatant per harvest was concentrated at 27,499 × *g* for 90 min at 4°C (Optima L-90K Ultracentrifuge, SW 32 Ti Swinging-Bucket rotor; Beckman Coulter). LVV pellets from each harvest were dissolved in 500 μL of PBS on ice for 30 min, carefully resuspended, and combined. Aliquots of the preparations were stored at −80°C. Modifications made to the scale of the preparations are indicated in the text.

### Cellular DNA extraction

DNA was extracted from cultured cells using the Qiagen DNeasy Blood and Tissue Kit with modifications described by Ingemarsdotter et al.^100^

### Preparation of cellular RNA and reverse transcription

Total cellular RNA was extracted using the Qiagen RNeasy Mini kit according to the manufacturer’s recommendations. Then 1 μg of RNA was treated with DNase using the TURBO DNA-free Kit (Thermo Fisher Scientific) according to the manufacturer’s routine or rigorous procedures. DNase-treated RNA (200 ng) was converted to cDNA as described by Ingemarsdotter et al.^100^ Control “no-RT” reactions were prepared without MultiScribe Reverse Transcriptase (RT; Thermo Fisher Scientific) to determine if DNase-treated RNA samples were free of detectable DNA.

### qPCR assays

qPCRs were performed on 7500 Fast or StepOnePlus Real-Time PCR Systems (Thermo Fisher Scientific). Thermal cycling conditions were as follows: 50°C for 2 min, 95°C for 20 s, and 40 cycles of 95°C for 3 s and 60°C for 30 s. Primer and probe sequences can be found in Table S2. Each 10-μL reaction was prepared in duplicate or triplicate. Experimental samples were loaded alongside negative, no-template controls (qPCR ctrl) in which water was added in lieu of DNA. For each assay, data ≥ 5 C_T_ values higher than the highest on-target C_T_ value were considered background amplification and excluded from the analysis. With this approach, C_T_ values considered to be background amplification ranged from 30 to undetectable across primer sets and assays, with the exception of one assay in which the lowest background C_T_ was 28 (Figure S6A).

#### Estimation of cell number by qPCR

Levels of *ALB*, a single-copy cellular gene (per haploid genome), were used to estimate the number of cells associated with a DNA sample. *ALB* qPCRs were prepared in 1× Fast SYBR Green Master Mix (Applied Biosystems) with 50–100 nM primer and 40- to 100-ng DNA template. A standard curve was prepared from 1:10 serial dilutions of DNA from control untreated cells (either Jurkat or HEK293T), with C_T_ values related to *ALB* copy number according to Stephenson.^101^ To estimate cell number, total *ALB* copy number was divided by the number of *ALB* gene copies in one cell: four in Jurkat based on karyotyping analysis and three in HEK293T based on previously published reports.^102^^,^^103^

#### Estimation of vector copy number by qPCR

Assays for HIV-1 cDNA copies (reverse transcripts) in transduced cells were based on the detection of HIV-1 *tat* exon 1 in cellular DNA, with qPCRs prepared in TaqMan Fast Advanced Master Mix with 37.5–50 nM primer, 100 nM probe, and 40–80 ng of DNA template. A plasmid standard curve was prepared with pNL4-3ΔE-EGFP or pNL4-3 (NIH AIDS Reagent Program), with C_T_ values related to gene copy number based on the formula described by Lee et al.^104^ and Barczak et al.^24^ Assays for therapeutic/control LVV cDNA copies (reverse transcripts) in transduced cells were based on the detection of the therapeutic/control-specific *WPRE* sequence, with qPCRs prepared in 1× TaqMan Fast Advanced Master Mix with 20–50 nM primer, 100 nM probe, and 40- to 100-ng DNA template. A plasmid standard curve was prepared with pSico.

To estimate the average VCN per cell following lentiviral transduction, *WPRE* (therapeutic/control vector) or *tat* (HIV-1) copy number was divided by the estimate for cell number (*ALB* qPCR).

#### Titration of infectious lentiviral particles by qPCR

TUs were approximated by LVV cDNA detected in cells transduced with the LVV preparations according to previously established methodology.^24^^,^^25^^,^^26^ Jurkat T cells (1-2 × 10^5^/well) were seeded in 24-well plates on day 1, counted and then transduced with 5 and 10 μL of LVV preparation (or mock; media) in triplicate wells on day 2 and lysed for cellular DNA extraction on day 5, whereby VCN was estimated by qPCR. The infectious titer of the LVV preparation was expressed as the average TU/mL of the six transductions (TDNs), with TU/mL calculated as follows^24^^,^^25^: $\frac{VCN\;x\;cells\;counted\;prior\;to\;TDN\;}{Lentivirus\;volume\;for\;TDN\;\left(\mu L\right)\times \frac{1\;mL}{1000\;\mu L}}$. As TU was approximated from VCN, here an MOI of e.g., 1 denoted an average of one vector cDNA copy (reverse transcript) per cell. Cells were counted prior to transduction with LVV preparations of known concentration to determine the LVV volume necessary for the intended MOI.

#### Estimation of lentiviral expression levels by RT-qPCR

Therapeutic and positive control LVV RNA payload levels were assessed by *HSVtk* qPCR following DNase treatment and reverse transcription of RNA extracted from transduced cells. Reactions were prepared in 1× TaqMan Fast Advanced Master Mix (Applied Biosystems) with 20–60 nM primer (Table S2), 100 nM probe, and cDNA equivalent to 20 ng of DNase-treated cellular RNA. A plasmid standard curve was prepared from 1:10 serial dilutions of CkRhsp-HSVtk or EF1α-HSVtk. Levels of HIV-1 *tat* exon 1 RNA, the target of therapeutic *trans*-splicing, were assessed by qPCR on cDNA equivalent to 20 ng of DNase-treated cellular RNA. *Tat* qPCRs were prepared and analyzed as earlier described.

*HSVtk* or *tat* copy number was then divided by the amount of template used in the qPCR (0.02 μg) to determine copies per microgram of total cellular RNA. Lentiviral expression levels were normalized to *β-actin* expression as per Ingemarsdotter et al.^100^ *β-actin* qPCRs were prepared in 1× Fast SYBR Green Master Mix (Applied Biosystems) with 40–50 nM primer and cDNA equivalent to 0.2–20 ng of DNase-treated total cellular RNA. A standard curve was prepared from 1:10 serial dilutions of TaqMan *β-actin* Template Reagents (Thermo Fisher Scientific) or pCAG-mGFP-actin (Addgene plasmid #21948).

### RT-PCR assay for chimeric HIV-1 *tat/HSVtk* RNA

The splice junction of *trans*-spliced RNAs was amplified by RT-PCR with one of two distinct primer pairs (Table S2), resulting in either a short amplicon (142 bp) or longer amplicon (291 bp). PCRs were prepared with 1.25 U of GoTaq DNA Polymerase (Promega), 1× GoTaq Buffer (Promega), 200 μM PCR nucleotide mix (Promega), nuclease-free water (Promega), 200 or 500 nM primer (for 142- or 291-bp amplicons, respectively), and cDNA equivalent to 50–80 ng of DNase-treated total cellular RNA. For 142-bp amplicons, thermal cycling conditions were as follows: 95°C for 2 min; 60 cycles of 95°C for 30 s, 55°C–60°C for 30 s, and 72°C for 30 s; 72°C for 5 min; and 4°C indefinite hold. For 291-bp amplicons, thermal cycling conditions were as follows: 95°C for 2 min; 40 cycles of 95°C for 30 s, 55°C for 30 s, and 72°C for 30 s; 72°C for 5 min; and 4°C indefinite hold.

For normalization, *β-actin* PCRs were prepared with the same buffer, DNA polymerase, and nucleotide specifications as *trans*-splice PCRs with the addition of 200 nM primer (Table S2) and cDNA equivalent to 10 ng of DNase-treated total cellular RNA. Thermal cycling conditions were as follows: 95°C for 2 min; 30 cycles of 95°C for 30 s, 60°C for 30 s, and 72°C for 30 s; 72°C for 5 min; and 4°C indefinite hold. PCR products were run on agarose gels with densitometry performed with ImageJ software.

Putative *trans*-spliced PCR products were isolated with the QIAquick Gel Extraction Kit (Qiagen) for Sanger sequencing (Eurofins Genomics) with the primers from the *trans*-splice assay (Table S2). Alternatively, low-copy PCR products were inserted into TOPO plasmid vectors for sequencing with TOPO-specific M13 primers (Table S2) by way of the TOPO TA Cloning Kit (Invitrogen). Chromatograms were visualized with SnapGene Viewer software, and BLAST was used to determine sequence identity.

### Western blots

HEK293T cells (5 × 10^5^/well) were seeded on day 1, transfected with 1 μg of lentiviral transfer plasmid (or mock; media) on day 3, and subjected to media change on day 4. On day 5, cells were washed with PBS and lysed in 200 μL of 1× Cell Culture Lysis Reagent (Promega) for 15 min. Lysed cells were then scraped and transferred to a microcentrifuge tube for clarification (1 min; 13,523 × *g*). Supernatant was transferred to a new microcentrifuge tube and treated with 1× Halt Protease Inhibitor Cocktail (Thermo Fisher Scientific). Protein concentration was determined with the Quick Start Bradford 1× Dye Reagent (Bio-Rad) according to the manufacturer’s recommendations for a microplate assay. Absorbances were measured on an iMark Microplate Reader (Bio-Rad) at 595 nm.

Denatured proteins (10 μg) were run on a 1.5-mm SDS-PAGE gel (10% [w/v] acrylamide resolving gel overlaid with 5% [w/v] acrylamide stacking gel) in 1× electrophoresis buffer (0.1 M Tris base, 0.38 M glycine, 0.1% [w/v] SDS) at 100–110 V in a Mini Protean Tetra Cell (Bio-Rad) for approximately 2 h. Proteins were transferred to 0.45-μm Amersham Protran Premium nitrocellulose membranes (SLS) in 1× transfer buffer (25 mM Tris base, 150 mM glycine, 10% [v/v] ethanol) at 100 V for 1 h on ice. A Mini Trans-Blot Cell (Bio-Rad) was used for the wet transfer. Membranes were blocked for 1 h at room temperature in PBS with 0.05% Tween 20 (PBST) with 4% (w/v) BSA prior to incubation with primary anti-HSV-1 thymidine kinase vN-20 goat polyclonal antibody (sc-28037; Santa Cruz Biotechnology) in staining buffer (PBST with 1.5% [w/v] BSA) overnight at 4°C. Membranes were then washed three times in PBST and incubated with secondary anti-goat rabbit polyclonal immunoglobulin/horseradish peroxidase (HRP) (P0449; Agilent) in staining buffer for 1 h at room temperature. Membranes were washed three times in PBST and developed with ECL Western Blotting Substrate (Promega) according to the manufacturer’s recommendations. Exposures were taken on the iBright FL1500 Imaging System (Thermo Fisher Scientific).

For normalization, membranes were stripped and re-probed for vinculin. Briefly, membranes were washed in PBS twice followed by a 20-min incubation at room temperature in Restore Western Blot Stripping Buffer (Thermo Fisher Scientific). Membranes were washed three times in PBS and then blocked for 1 h at room temperature, followed by incubation overnight at 4°C with primary anti-vinculin recombinant rabbit monoclonal antibody (42H89L44; Invitrogen) in staining buffer. Membranes were then washed as previously described and stained for 1 h at room temperature with secondary HRP-conjugated anti-rabbit goat IgG (H + L) antibody (Invitrogen) in staining buffer. Membranes were washed, developed, and exposed as previously described.

### MTT cell-viability assay

The MTT assay for cell viability was undertaken as described by Ingemarsdotter et al.^16^ For suspension cells, round-bottom 96-well plates (Corning) were used so formazan crystals could be pelleted by centrifugation (935 × *g* for 5 min). Supernatant was then removed, taking great care to avoid disturbing the pellet. Absorbance (Abs) was measured on an iMark Microplate Reader, with the 655-nm background reading subtracted from the 595-nm reading. Viability scores were calculated relative to control (mock treatment/transduction) cell absorbance as follows: ${\%Viability}_{experimental}=\frac{{Abs}_{experimental}}{{Abs}_{control}}\times 100$. The viability of control cells was set to 100%.^16^ To estimate the percentage of LRA-stimulated, LVV-transduced cells that were effectively eliminated with GCV, the following calculation was performed:

$$100-\left[\left(\;\frac{\%\;Viability\;of\;cells\;subjected\;to\;LRA+LVV+GCV}{\%Viability\;of\;cells\;subjected\;to\;LRA+LVV}\right)\times 100\right]\text{.}$$

### HIV-1 reactivation assay

To determine the optimal dose of LRA to use with the HIV-1 RNA-targeted CSS, the design of reactivation assays was modeled on the assay that would test the two in combination for shock and kill. J-Lat 10.6 and Jurkat T cells (GFP− control) were seeded at 5 × 10^3^/well on day 1, treated with LRAs (or mock; media) on day 2 or 3, and analyzed on day 8. All LRA stocks were diluted in media rather than the original solvent prior to addition to cells. Diluent (media)-only treatments were performed in place of LVV transduction (day 2 or 3) and ganciclovir treatments (days 4 and 5) to align with assays in which the HIV-1 RNA-targeted CSS was used alongside LRAs.

For flow cytometry, cells in one well per condition were stained with DRAQ7 (1:1,000 in PBS; Abcam) on ice for 5 min in the dark. Cells were then washed with PBS-0.1% (w/v) BSA and resuspended in PBS for immediate analysis on an Accuri C6 Flow Cytometer (BD Biosciences). DRAQ7 was excited by the 640-nm line with emissions detected in FL4 (675/25-nm optical filter). Cellular EGFP was excited by the 488-nm line with emissions detected in FL1 (533/30-nm optical filter). Data were analyzed with FlowJo 10 software, whereby lymphocytes were first identified and gated according to their forward and side scatter properties. Doublets and other debris were excluded from the analysis based on their area and height. Control, unstained J-Lat 10.6 cells were used to determine the DRAQ7− gate and the parental Jurkat T cell line was used to determine the GFP− gate. HIV-1 reactivation was expressed as the percentage of live (DRAQ7−) lymphocytes that were GFP+. Dose-response curves were fitted by nonlinear regression with GraphPad Prism 9 and were used to estimate the relative half-maximal effective concentration (EC_50_) of each LRA. Unstimulated J-Lat 10.6 cells were included in each experiment to determine the baseline level of HIV-1 reactivation without LRA treatment (GFP+).

To assess the effect of LRA treatment on the growth of cell-culture populations, in select experiments one or two wells per condition were additionally subject to the MTT assay for cell viability as described above.

### Statistical analysis

Analyses were performed in GraphPad Prism 9 with an alpha of 0.05. Error bars represent the standard deviation (SD) from the mean. For comparisons between two groups, a two-tailed unpaired t-test was performed. For comparisons involving more than two groups, one-way ANOVA with Tukey’s multiple comparisons test was performed. ∗*p* < 0.05, ∗∗*p* < 0.01, ∗∗∗*p* < 0.001.

## Data and code availability

The data that support the findings of this study are available within the article and supplementary materials and from the corresponding author, A.B.B., upon reasonable request. The shuffle-and-fold script is available at https://gitlab.com/FinKM/rna-shuffle-fold.

## Acknowledgments

We would like to thank Hoi-Ping Mok, Ulrich Desselberger, John Sinclair, Mike Malim, Mark Wills, Bo Meng, Julia Kenyon, Nyarie Sithole, Nicholas Norton, Emma Poole, Benjamin Krishna, and Sarah Jackson for helpful discussions. We are grateful to Harriet Groom, Gennaro Iaconis, and Isobel Jarvis for advice and training at containment level 3. This research was supported by the Cambridge NIHR BRC Cell Phenotyping Hub. We would also like to acknowledge the Cytogenetics Laboratory (Medical Genetics Service) at Cambridge University Hospitals for karyotyping services and the Research Instrumentation and Cell Services core facility at Cancer Research UK (Cambridge Institute) for mycoplasma testing services.

The reagents pMDLg/pRRE and pRSV-Rev were obtained through Addgene from Didier Trono. The reagents pSico, pCMV-VSV-G, pEF-GFP, and pCAG-mGFP-actin were obtained through Addgene from Tyler Jacks, Bob Weinberg, Connie Cepko, and Ryohei Yasuda, respectively. Through the AIDS Research and Reference Reagent Program, Division of AIDS, NIAID, NIH the reagent pNL4-3 was obtained from Malcolm Martin and pNL4-3ΔE-EGFP was obtained from Haili Zhang, Yan Zhou, and Robert Siliciano. We apologize to the colleagues whose work could not be referenced due to space restrictions.

This work was supported by a 10.13039/501100000265Medical Research Council Confidence in Concept award (RCAG/697) and a Higher Education Funding Council for England award via the Stevenage Bioscience Catalyst (RCAG/655). Personal support was received from Peterhouse (to A.B.B.) and the Clinical Academic Reserve (to A.M.L.L.).

## Author contributions

Conceptualization, A.B.B., C.K.I., and A.M.L.L.; methodology, A.B.B. and C.K.I.; investigation, A.B.B., S.H., and C.K.I.; software development, F.K.-M.; writing – original draft, A.B.B.; writing – review & editing, A.B.B., C.K.I., and A.M.L.L.; funding acquisition, C.K.I. and A.M.L.L.; supervision, C.K.I. and A.M.L.L.

## Declaration of interests

C.K.I. and A.M.L.L. are inventors on a patent application relating to parts of this work and have management roles with shareholdings in Spliceor Ltd. A.B.B. formerly consulted for Spliceor Ltd. F.K.-M. is an employee of Cambridge Design Partnership (Cambridge, UK) but contributed to the work exclusively in a personal capacity. S.H. contributed to the work while affiliated exclusively with the University of Cambridge but is now an employee of Gerson Lehrman Group.

## Supplemental information

Document S1. Figures S1–S12 and Tables S1 and S2

Document S2. Article plus supplemental information

## Footnotes

Supplemental information can be found online at https://doi.org/10.1016/j.omtn.2024.102341.
