# Supplementary material for: Optimization of a lentivirus-mediated gene therapy targeting HIV-1 RNA to eliminate HIV-1-infected cells
Source: Mol Ther Nucleic Acids. 2024 Sep 16;35(4):102341. doi: 10.1016/j.omtn.2024.102341 (PMC11491724; doi:10.1016/j.omtn.2024.102341)
Supplement: Document S1. Figures S1–S12 and Tables S1 and S2 [file mmc1.pdf]

OMTN, Volume *35*

Supplemental information

Optimization of a lentivirus-mediated gene therapy targeting HIV-1 RNA to eliminate HIV-1-infected cells

Amanda B. Buckingham, Sophia Ho, Finlay Knops-Mckim, Carin K. Ingemarsdotter, and Andrew M.L. Lever

SUPPLEMENTAL FIGURES

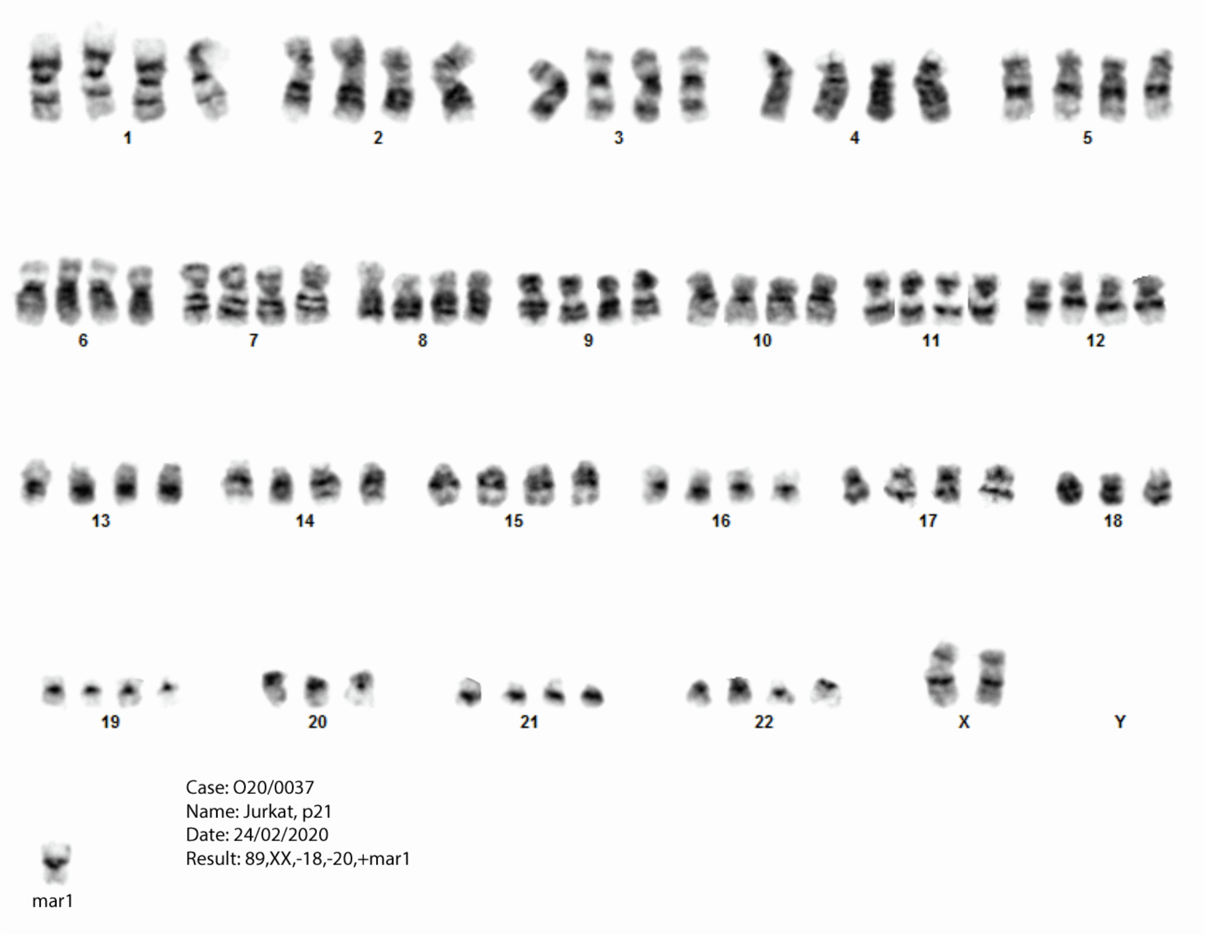

<u>**Figure S1**</u>

G-banded karyotype of Jurkat T cells. Jurkat T cells were karyotyped so that the number of cells associated with a sample of cellular DNA could be estimated from the number of copies of a target cellular gene, as determined by qPCR. Karyotyping and analysis were performed by the Cytogenetics Laboratory (Medical Genetics Service) at Cambridge University Hospitals. All cells (N = 9) were determined to be near tetraploid with chromosome counts of 86 to 89. Representative spread depicted.

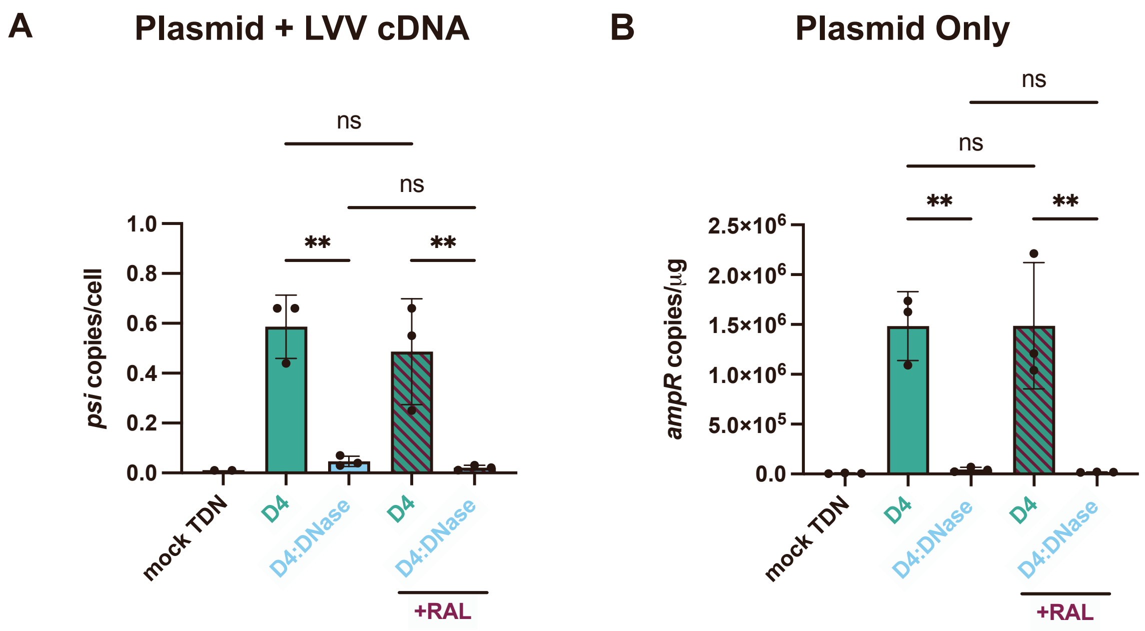

<u>Figure S2</u>

Persistence of Lentiviral Plasmids in LVV Preparations Not treated With Benzonase. CkRhsp-directed D4 was prepared according to the original LVV production protocol, in which plasmid carryover was not controlled. The effect of two different treatments on the persistence of lentiviral DNA (transfer plasmid and LVV cDNA) in transduced cells was then explored: Benzonase endonuclease, to antagonize plasmid DNA exclusively, and the HIV-1 integrase inhibitor raltegravir (RAL), to antagonize LVV cDNA exclusively. An aliquot of the LVV preparation was first split into two fractions, with one treated with Benzonase at 37°C for 15 min and the other left untreated, prior to freezing at -80°C. 2 x 10^5 Jurkat cells/well were seeded on day 1, treated with 500 nM RAL or media (mock control) for 4 hr prior to transduction with 10 µL D4 LVV (either Benzonase-treated or untreated) on day 2, and lysed for DNA extraction on day 8. (**A**) Combined levels of transfer plasmid and LVV cDNA per cell, assessed by *psi* qPCR on DNA extracted from transduced cells with *ALB* qPCR used for normalization. (**B**) Levels of lentiviral plasmid (per µg total DNA) that co-purified with cellular DNA following LVV transduction, assessed by *ampR* qPCR normalized to factor difference in *ALB* levels from mock transduced cells. (*A-B*) Data presented as mean with SD (N = 3 wells/condition). ** $P < 0.01$; one-way ANOVA with Tukey's multiple comparisons test.

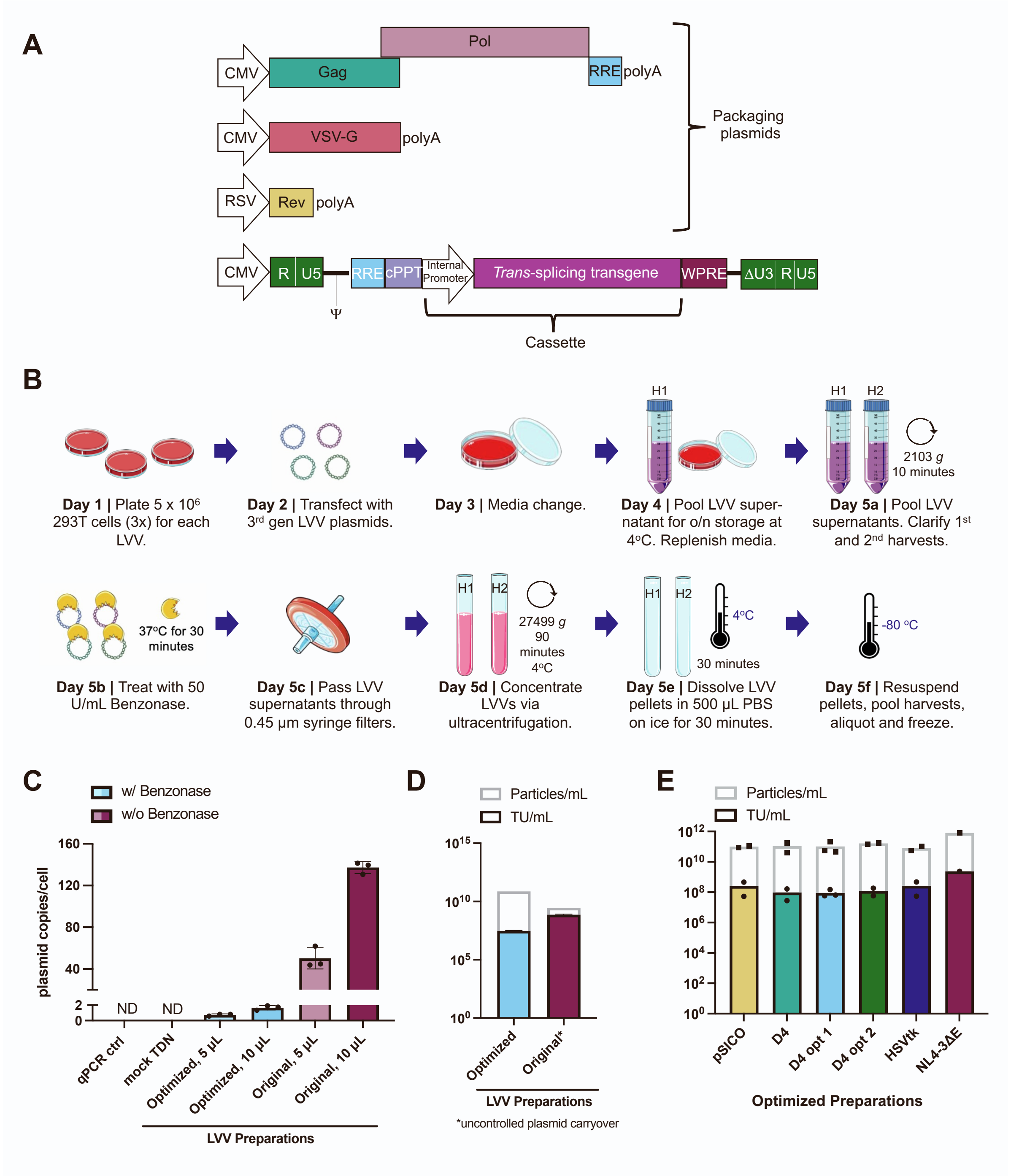

Figure S3

Optimization of Lentivirus Production. (**A**) Schematic of VSV-G-pseudotyped third-generation,[1] self-inactivating[2] LVV system for delivery of the HIV-1-targeting payload. CkRhsp[3] or EF1α[4] used as internal promoter for payload expression. Plasmids additionally expressed *ampR* selection marker. (**B**) Workflow for LVV production by transient transfection, optimized for high infectious titer and minimal plasmid carryover. See Materials and Methods, main text, for further details. LVV, lentiviral vector; H1, first harvest; H2 second harvest; o/n, overnight. Figure created with elements from Servier Medical Art (Creative Commons Attribution 3.0 Unported License, www.smart.servier.com). (**C**) Levels of lentiviral plasmid that co-purified with cellular DNA following transduction with LVV, assessed with *ampR/ALB* qPCRs. Jurkat T cells were seeded at 2 x 10^5/well on day 1, transduced with LVV (5, 10 µL) prepared with the original or optimized methods on day 2, and subjected to DNA extraction on day 5.

Data presented as mean with SD (N = 3 transductions). ND, below limit of detection. (**D**) Influence of uncontrolled plasmid carryover on estimate for infectious titer (Jurkat transducing units, TU/mL), assessed with *psi/ALB* qPCRs for vector copy number (VCN) on DNA from (*C*). Total LVV particles/mL estimated by HIV-1 p24 capsid ELISA on inactivated LVV aliquots. Data presented as mean TU/mL with SD (N = 6 transductions; 5 and 10 μL in triplicate). (**E**) Infectious titer (Jurkat TU/mL) of lentivirus preparations made according to the optimized method, assessed with *WPRE* (for therapeutic and positive control LVVs; and pSico, empty LVV) or *tat* (for HIV-$1_{NL4\text{-}3\Delta E}$) qPCRs normalized to *ALB* qPCR on DNA from Jurkat cells transduced as in (*C*). Total lentivirus particles/mL estimated as in (*D*). Data (circles, TU/mL; squares, particles/mL) represent concentration of independent preparations, N ≥ 2 with exception of HIV-$1_{NL4\text{-}3\Delta E}$. Optimized method scaled up by a factor of two for HIV-$1_{NL4\text{-}3\Delta E}$. CkRhsp-directed LVV depicted.

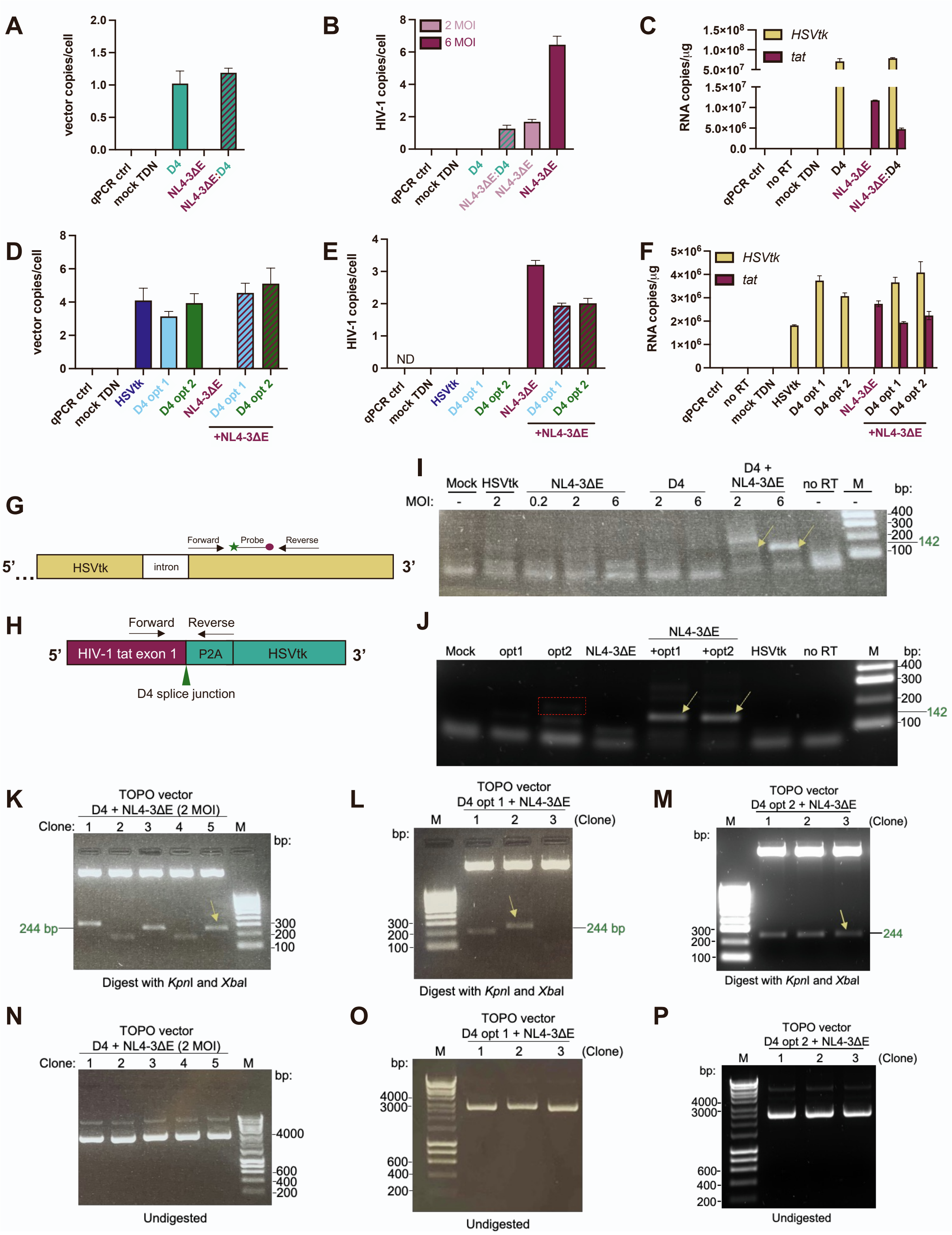

Figure S4

Validation of HIV-1 RNA-targeting LVVs in an HIV-1-expressing T-cell Line. (**A-P**) Jurkat T cells were seeded in duplicate at 1 x 10^5/well on day 1, transduced with HIV-$1_{NL4\text{-}3\Delta E}$ (or mock; media) on day 2, transduced with CkRhsp-driven therapeutic or control LVV (or mock; media) on day 3, and subjected to either DNA or RNA extraction on day 5. (*A-C, I, K, N*) MOI = 2 unless otherwise specified. (*D-F,J,L-M,O-P*) MOI = ~4. (*A,D*) VCN assessed by qPCR for *WPRE* on DNA from transduced Jurkat

cells, normalized to *ALB* levels. (*A*) D4 LVV was produced with some reductions in scale. (*B,E*) HIV-$1_{NL4\text{-}3\Delta E}$ cDNA copies per cell assessed by qPCR for *tat* on DNA from transduced Jurkat cells, normalized to *ALB* levels. (*C,F*) Levels of therapeutic/positive control RNA payload and HIV-$1_{NL4\text{-}3\Delta E}$ RNA target (per µg total cellular RNA) in transduced Jurkat cells, assessed by RT-qPCR for *HSVtk* and *tat,* respectively, normalized to factor difference in *β-actin* expression from mock transduced cells. (*A-F*) Data presented as mean with SD (N = 2 or 3 qPCR replicates). (*G*) Primer and probe design for *HSVtk* qPCR, developed by Poddar *et al.*[5] Target sequence present in both positive control and therapeutic payloads, regardless of *trans*-splicing. (*H*) PCR primer design for amplification of the splice junction of chimeric HIV-1/*HSVtk* transcripts (short amplicon), with the forward primer positioned in HIV-1 *tat* exon 1 and the reverse positioned in the p2A self-cleaving peptide sequence in the therapeutic payload. Diagram not to scale. (*I-J*) RT-PCR detection of putative chimeric HIV-1/*HSVtk* RNA sequences (142 bp; yellow arrows) in HIV-1-expressing Jurkat T cells following delivery of (*I*) D4, (*J*) D4 opt 1 or D4 opt 2 therapeutic LVV. Nonspecific PCR products also were detected, including a 166 bp amplicon (see *J*, boxed in red) that was found to be indicative of off-target therapeutic *trans*-splicing (see Figure S5 for characterization). PCR products were cloned into TOPO plasmids for sequencing. M, marker. (*K-M*) *Kpn*I and *Xba*I restriction digest screen of sequencing plasmids for putative HIV-1/*HSVtk trans*-spliced PCR product insert (244 bp with addition of TOPO sequences) from HIV-1-expressing Jurkat cells transduced with (*K*) D4, (*L*) D4 opt 1, or (*M*) D4 opt 2. A subset (yellow arrow) was validated by sequencing (see Figure 2B-E). (*N-P*) Mock reaction of sequencing plasmids without restriction enzymes as negative control. TOPO cloned PCR products from (*L,O*) derived from *trans*-splice RT-PCR (data not shown) with 45 amplification cycles instead of 60, using RNA from Jurkat cells treated under identical experimental conditions.

A

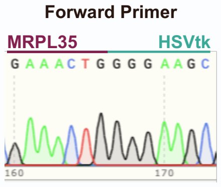

B **Extracted chromatogram sequence:**

TCCTATGGCAGGAAGAAGCGATTGAGGGAATTTGTATTCTGCAATAAAACCCA
GAGTAAACTCTTAGATAAAATGACGACGTCCTTCTGGAAGAGGCGAAACTGGG
GAAGCGGAGCTACTAACTTCAGCCTGCTGAAGCAGGCTGGAGACGTGGAGGA
GAACCCTG

C

Extrapolated *MRPL35*/*HSVtk* fusion sequence:

AUGgcugccucugccuuugcuggugcagugagagcagcuucaggaauccuacggccccugaauauuuggcaucuucaaccuaccgcaacugugucaag
aaugccucucuuauuucugcauuguccacuggacguuuuagucauauucagacaccaguuguuuccuccacucccagacuuaccacaucugagagaaacc
ugacaugugggcauaccucagugauccuuaauagaauggcccccgugcuuccaaguguccugaagcugccagucagaucucuaacauacuucagugcaag
aaaaggcaagagaaagaccgugaaagcugucaucgauagguuucuucgacuucauuguggccuuugggugaggagaaaggcuggcuauaagaaaaaauu
auggaaaaagacaccugcaaggaagaagcgauugagggaauuuguauucugcaauaaaacccagaguaaacucuuagauaaaaugacgacguccuucug
gaagaggcgaaacugggaagcggagcuacuaacuucagccugcugaagcaggcuggagacguggaggagaacccugggccu*GCU*...

From AUG to start of *HSVtk*$_{\Delta AUG1}$ (inclusive) = 582 bases/3 = 194 codons. *HSVtk* would be in-frame with respect to *MRPL35* start codon.

Formatting	Meaning
Underline	PCR primer
Purple highlight	PCR product aligning to *Homo sapiens MRPL35*, transcript variants 1 and 3, mRNA (NCBI Reference Sequences: NM_016622.4 and NM_001363782.1)
Purple text	Continuation of *MRPL35* (extrapolated from Ensembl transcripts for ENSG00000132313)
**BOLD CAPS**	*MRPL35* start codon
Turquoise highlight	PCR product aligning to CkRhsp-directed D4 opt 2 payload
Turquoise Text	Continuation of CkRhsp-directed D4 opt 2 payload sequence
ITALIC CAPS	Start of *HSVtk*$_{\Delta AUG1}$

Figure S5

Detection of chimeric cellular/*HSVtk* mRNA in Jurkat T cells following LVV-mediated delivery of CkRhsp-directed therapeutic payload. Analysis of 166 bp RT-PCR product from Jurkat T cells transduced with CkRhsp-directed D4 opt 2 LVV (Figure S4J), which was purified by gel extraction, cloned into a TOPO plasmid, and sequenced with M13 TOPO primers. Refer to Figure S4 for full experimental details. (**A**) Chromatogram snapshot of RT-PCR product. (**B**) Full sequence of RT-PCR product. (**C**) Analysis of RT-PCR product. Using BLAST alignments and the Splice Site Prediction by Neural Network tool,[6] the chromatogram sequence was identified as the product of an off-target *trans*-splicing reaction between *Homo sapiens* mitochondrial ribosomal protein L35 (*MRPL35*) donor exon 4 [splice variant *201* or *203* (Ensembl release 109, gene ENSG00000132313, transcript ENST00000254644.12 or ENST00000-409180.1; https://www.ensembl.org)] and the *HSVtk*$_{\Delta AUG1}$ acceptor exon. To understand if *HSVtk*$_{\Delta AUG1}$ would gain an in-frame start codon from the reaction, the sequence 5' of that amplified by PCR was extrapolated from Ensembl transcript data for *MRPL35*. *MRPL35* transcripts shared no sequence identity with the HIV-1 D4-targeting binding domain based on BLAST alignments, suggesting that *MRPL35* was not additionally targeted and that *trans*-splicing with *HSVtk*$_{\Delta AUG1}$ had been a stochastic event.

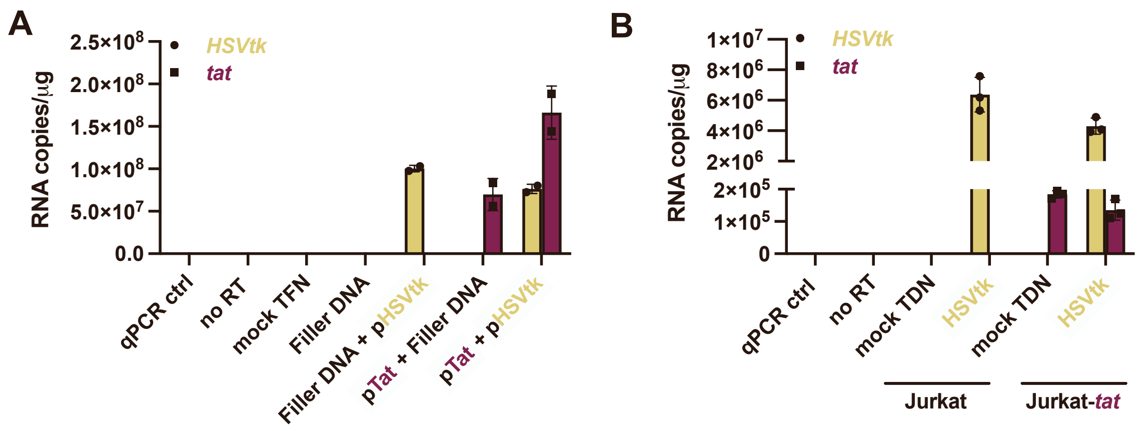

Figure S6

HIV-1 Tat Does Not Enhance CkRhsp-driven *HSVtk* RNA Levels. (**A-B**) Estimated *HSVtk* RNA payload levels relative to HIV-1 *tat* expression (per µg total cellular RNA), assessed by RT-qPCR for *HSVtk* and *tat*, respectively, normalized to *β-actin.* (*A*) 5 x 10^5 HEK293T cells/well were seeded on day 1, transfected with 500 ng pcDNA-tat plasmid (pTat) or pBluescript (filler DNA) on day 2, transfected with 500 ng CkRhsp-driven *HSVtk* in pVAX-1 (pHSVtk) or filler on day 3, subjected to media change on day 4, and lysed for RNA extraction on day 5. *HSVtk* was expressed from the pVAX-1 plasmid in lieu of the pSico transfer plasmid (Addgene plasmid #11578) to avoid the confounding effect of the promoter for LVV genome expression (split CMV/5'LTR) positioned upstream of the transgene cassette in pSico. Data presented as mean with SD (N = 2 wells/condition). (*B*) 5 x 10^4 Jurkat and Tat-expressing Jurkat-*tat* cells[7,8]/well were seeded on day 1, transduced with CkRhsp-driven HSVtk LVV (MOI = 8.3) on day 2, and subjected to RNA extraction on day 6. HSVtk lentivirus was produced with some reductions in scale. HIV-1 Tat levels in Jurkat-*tat* cells were previously established to be sufficient for *trans*-activation of the HIV-1 5'LTR.[8] Data presented as mean with SD (N = 3 wells/condition). (*A-B*) Mock treatments performed with media.

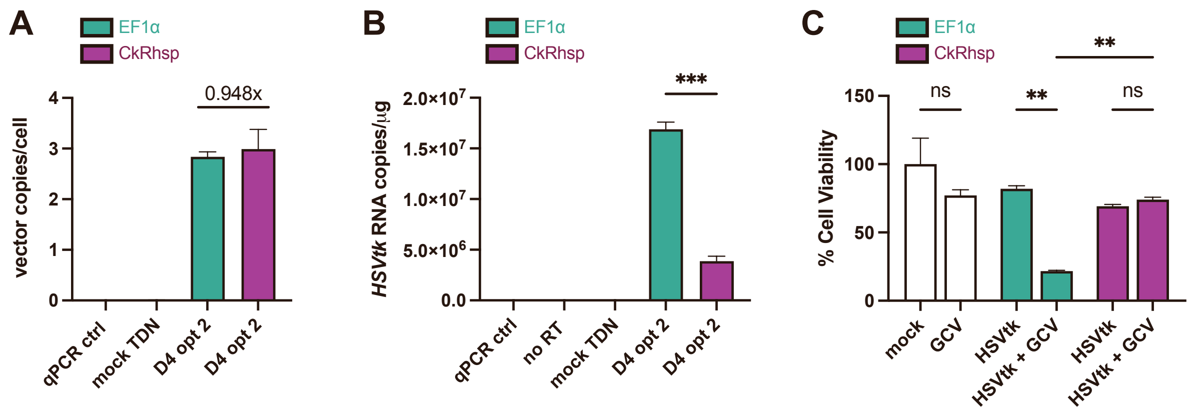

<u>**Figure S7**</u>

Replacement of CkRhsp with EF1α Promoter Enhances Transgene Expression. (**A**) LVV cDNA copies per cell (VCN), assessed by qPCR for *WPRE* on DNA from transduced Jurkat cells normalized to *ALB* levels. Data presented as mean with SD (N = 3 qPCR replicates). (**B**) Levels of therapeutic RNA payload (per µg total cellular RNA) in transduced Jurkat cells, assessed by RT-qPCR for *HSVtk* normalized to *β-actin* expression. Data presented as mean with SD (N = 3 wells/condition). *** P < 0.001; two-tailed unpaired t-test. (*A-B*) 1 x 10^5 Jurkat cells/well were seeded on day 1, transduced with EF1α- or CkRhsp-driven D4 opt 2 LVV (MOI = 4) on day 2, and lysed for DNA or RNA extraction on day 5. (**C**) Viability screen in uninfected cells with full-length HSVtk positive control and GCV. 5 x 10^3 Jurkat cells/well were seeded on day 1, transduced with EF1α- or CkRhsp-driven HSVtk LVV (MOI = 4) on day 3, treated with 50 µM GCV doses on days 4 and 5; and subjected to MTT cell viability assay on day 8. Cells were unaffected by GCV when transduced with CkRhsp-directed HSVtk at an MOI of 4, though were GCV-sensitive at an MOI of 14 (Figure 2G), suggesting that there was insufficient *HSVtk* payload at the lower concentration for cell death to be induced. Data presented as mean with SD (N = 2 or 3 wells/condition). ** P < 0.01; one-way ANOVA with Tukey's multiple comparisons test. (*A-C*) Mock treatments performed with media.

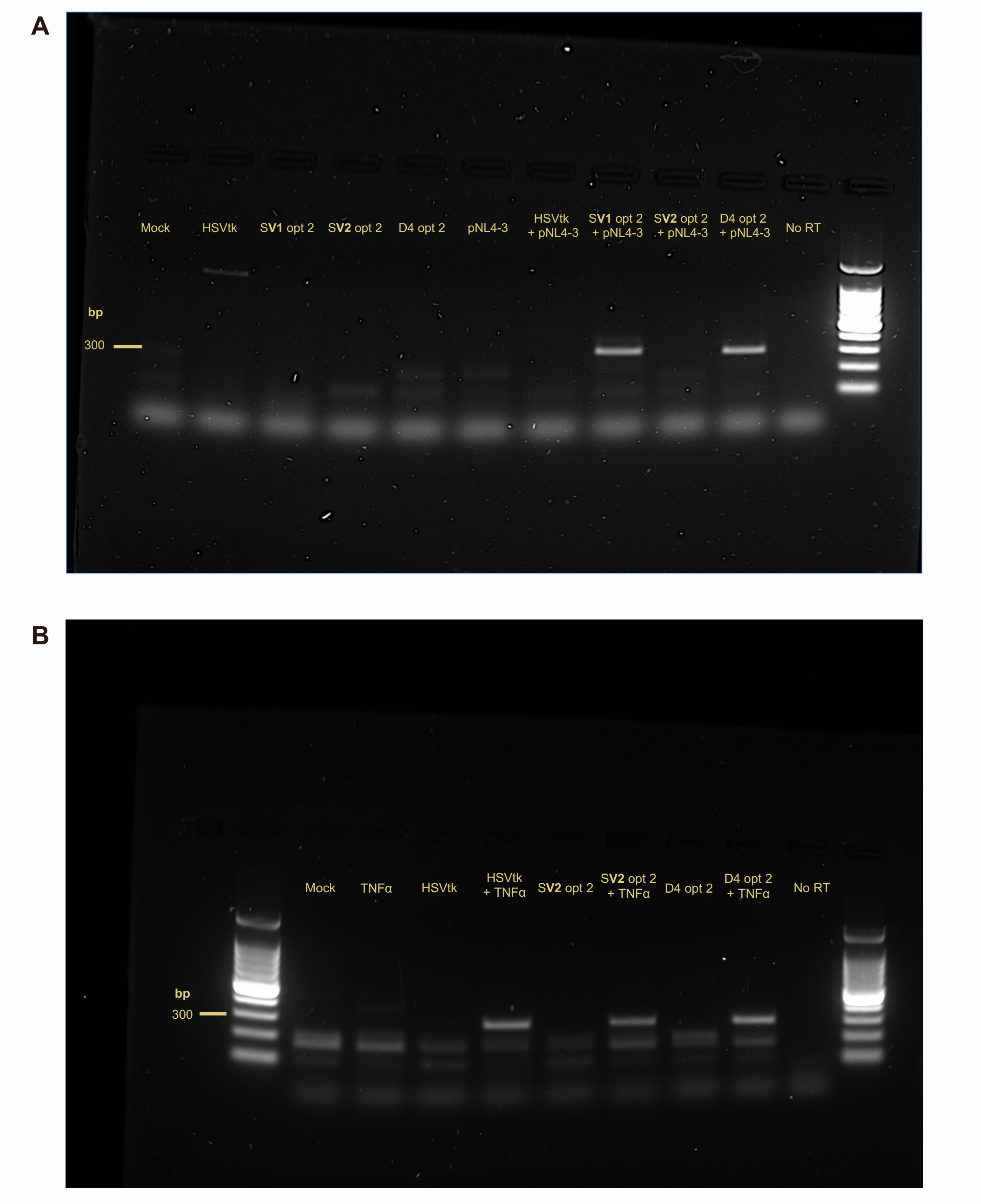

__Figure S8__

Electrophoresis gels of RT-PCR assays for HIV-1/*HSVtk* chimeric transcripts. (**A-B**) Uncropped images used to create (*A*) Figure 4C and (*B*) Figure 6C.

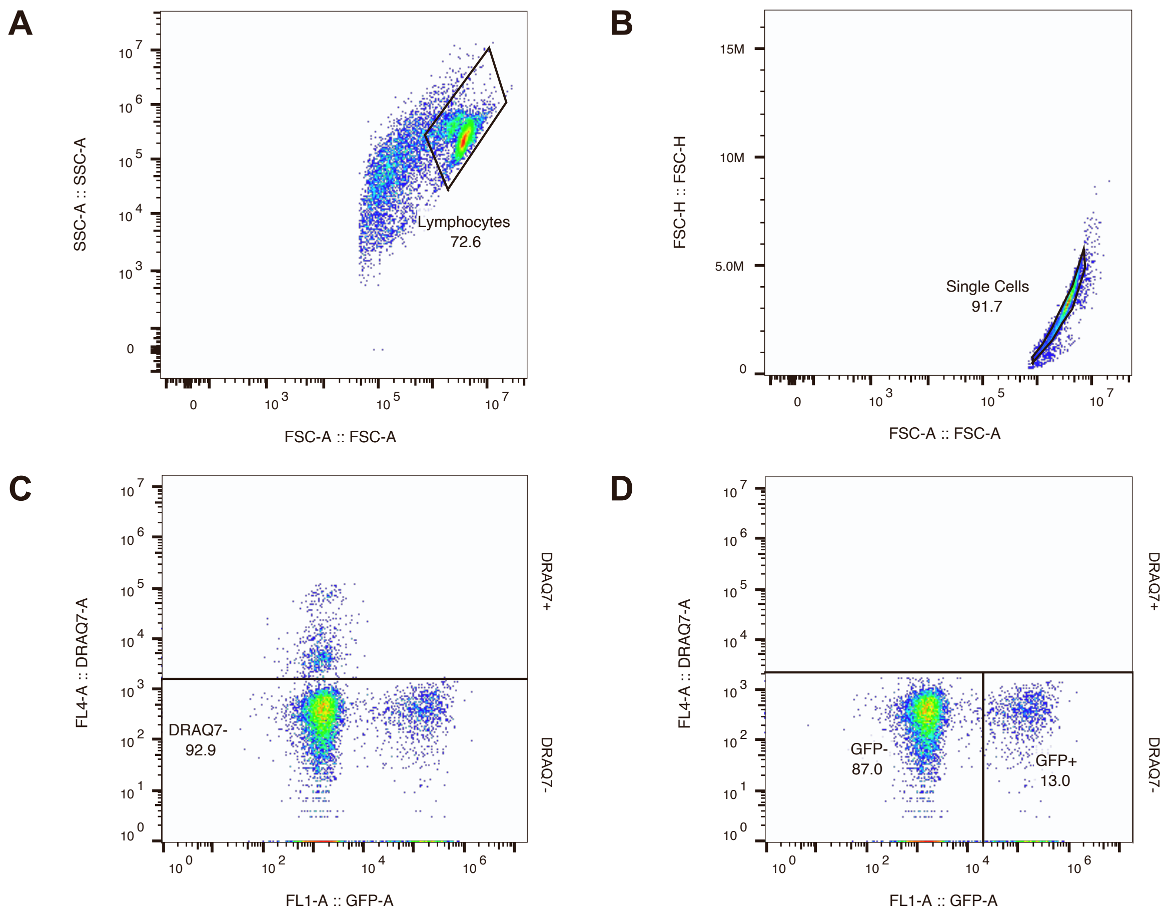

Figure S9

Gating strategy used to assess HIV-1 reactivation in J-Lat 10.6 cells. Example flow plots from J-Lat 10.6 cells reactivated with 300 pM PMA; refer to Figure 5B for details of reactivation assay. (**A**) Identification of lymphocytes based on forward and side scatter. (**B**) Identification of single cells within the lymphocyte population through exclusion of doublets and debris of disproportionate area relative to height. (**C**) Division of single-cell lymphocyte population into live (DRAQ7-) and dead (DRAQ7+) sub-populations. Unstained J-Lat 10.6 cells were used to establish the DRAQ7- cutoff in each assay (data not shown). (**D**) Division of live (DRAQ7-), single-cell lymphocyte population into GFP- and GFP+ sub-populations. The Jurkat parental cell line, which does not express *EGFP*, was used to establish the GFP- cutoff in each assay (data not shown).

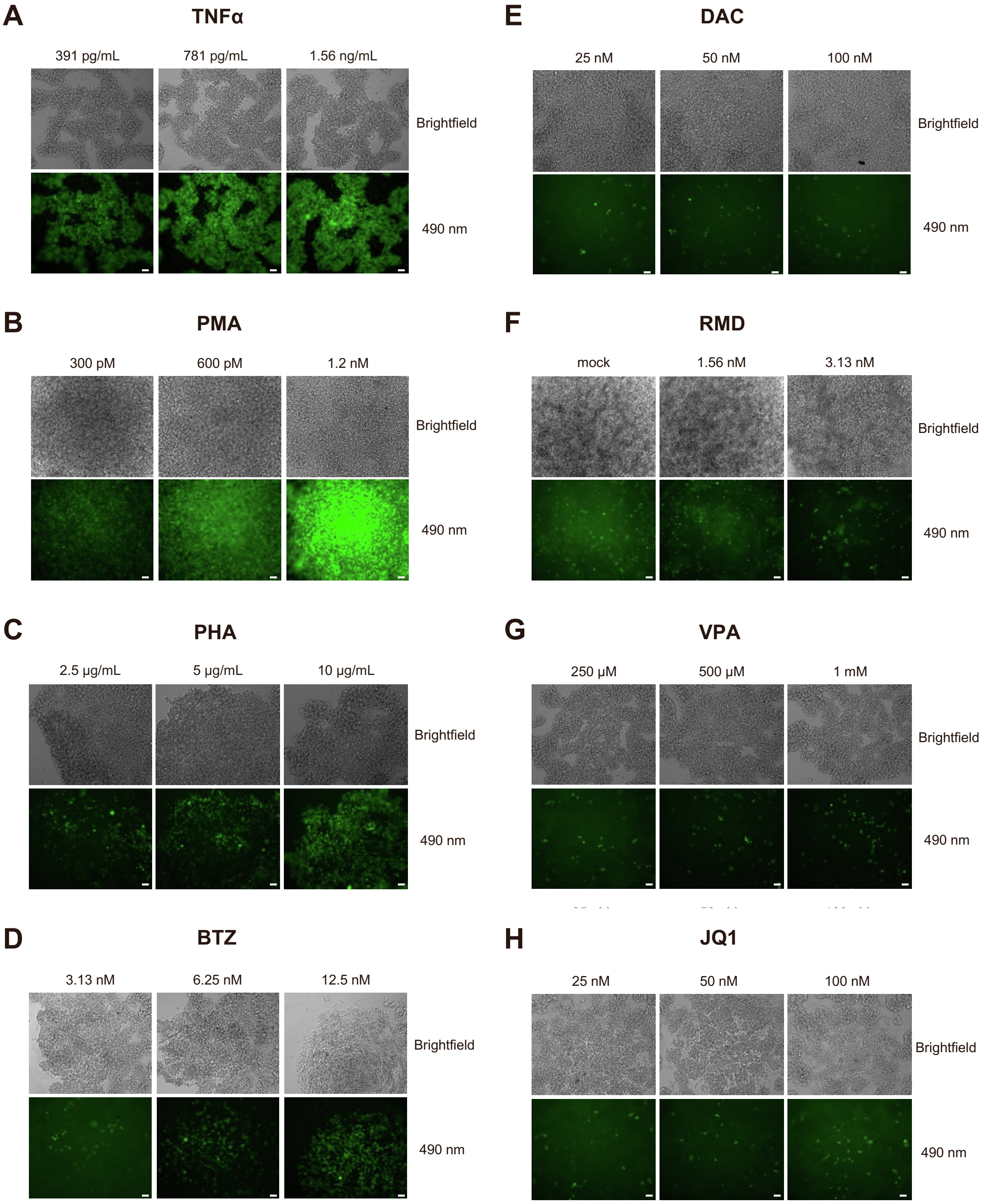

Figure S10

Dose-response of Chronically HIV-1-infected J-Lat 10.6 Cells to LRA Treatment, as Illustrated by Fluorescence Microscopy. Live-cell fluorescence and brightfield images were taken (**A**, **C**, **E**, **G**, **H**) 24 hr, (**D**) 48 hr, or (**B**, **F**) six days post-LRA treatment, illustrating EGFP expression levels at the specified concentration and time point. Fluorescence images do not necessarily capture the maximum level of EGFP expression that occurred over the time course. Scale bar indicates ~50 µm. Refer to Figure 5 for experimental details.

A Forward Primer

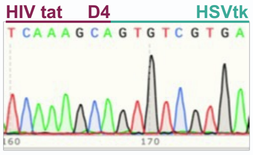

B **Extracted chromatogram sequence:**

AACCGGGGCTTACTGCTTGTACAATTGCTATTGTAAAAGTGTTGCTTTCATTGC
CAAGTTTGTTTCATGACAAAAGCCTTAGGCATCTCCTATGGCAGGAAGAAGCG
GAGACAGCGACGAAGAGCTCATCAGAACAGTCAGACTCATCAAGCTTCTCTAT
CAAAGCAGTGTCGTGAGCTAGCATATTGTGCTTCCACCACTGCCAATAACAAA
ATAACTAGCCACCATGGCTTCGTACCCCTGCCATCAACACGCGTCTGCGAA

C

Extrapolated HIV-1/*HSVtk* fusion sequence:

...GAGGAGAGCAAGAA**AUG**GAGCCAGUAGAUCCUAGACUAGAGCCCUGGAAGCAUCCAGGAAGUCAGCCUAAAacug
cuuguaccaauugcuauuguaaaaaguguugcuuucauugccaAGuuuguuucaugacaaaAGccuuAGgcaucuccuauggcaggaagaagcggag
acagcgacgaagagcucaucagaacagucagacucaucaagcuucucuaucaaagcagugucgugagcuagcauauugugcuuccaccacugccaauaac
aaaauaacuagccacc*AUG*gcuucguaccccugccaucaacacgcgucugcg...

Formatting	Meaning
Underline	PCR primer
Purple highlight	Segment of PCR product with 156/158 nt alignment to *tat* exon 1 of Mutant HIV-1 isolate J-Lat10.6_HIV, complete genome (GenBank: MN989412.1)
Yellow highlight	Corrected HIV-1 sequence (based on MN989412.1) assuming gaps in PCR product are sequencing artifacts
Purple text	Continuation of *tat* exon 1 (extrapolated from MN989412.1)
**BOLD CAPS**	*tat* exon 1 start codon
Yellow text	Overlapping A4 splice site cluster (5'-3': A4c, A4a, A4b) with AG dinucleotide sequence of each site in capital letters
White text	*EF1α* exon 2 (truncated/non-coding)
Turquoise highlight	Segment of PCR product aligning to truncated exon 2 of EF1α promoter, continuing into full-length *HSVtk* payload
ITALIC CAPS	Start of full-length *HSVtk*

D

Position (5'-3')	ORF	Frame	Kozak Sequence	Consequence for HSVtk	Resultant Polypeptide
1	HIV-1 *tat 1*	1	CAAGAA**AUGG**	Out of frame with *tat* exon 1	*Tat1*-novel fusion
2	HIV-1 *rev 1*	2	UCUCCU**AUGG**	Not translated (premature stop codon)	*Rev1*-novel fusion
3	*HSVtk*	2	GCCACC**AUGG**	Translated as intended	Full-length HSVtk

Consensus Kozak Sequence GCCRCC**AUGG**

Figure S11

Detection of chimeric HIV-1/*HSVtk* mRNA in TNFα-stimulated J-Lat 10.6 cells following LVV-mediated delivery of EF1α-directed full-length *HSVtk* payload. Analysis of ~280 bp RT-PCR product from TNFα-stimulated J-Lat 10.6 cells transduced with HSVtk LVV (Figure 6C), which was purified by gel extraction and sequenced with *trans*-splice PCR primers. Refer to Figure 6 for full experimental details. (**A**) Chromatogram snapshot of RT-PCR product. (**B**) Full sequence of RT-PCR product. (**C-D**) Analysis of RT-PCR product. (*C*) Characterization. Through BLAST analysis, the chromatogram sequence was characterized as a fusion between HIV-1 *tat* exon 1 and truncated *EF1α* exon 2, a noncoding 9 nt sequence that would be appended to the 5' end of the EF1α-directed full-length *HSVtk* RNA payload during transcription. Using the Splice Site Prediction by Neural Network tool,[6] we determined that the chimera was the product of RNA *trans*-splicing between HIV-1 D4 and a splice acceptor positioned at the 3' terminus of *EF1α* intron 1, proximal to the truncated exon 2. Intron 1 was included in the EF1α promoter region of the LVV transgene as it was found in a prior report to enhance

transcriptional activity.[4] The sequence 5' of that amplified by PCR was extrapolated from the sequence of the HIV-1 provirus in J-Lat 10.6[9] (GenBank: MN989412.1). The HIV-1_{HXB2} A4 splice site cluster was identified with reference to Sertznig *et al*.[10] (*D*) Identification and analysis of ORFs in extrapolated HIV-1/*HSVtk* mRNA sequence. The consensus Kozak sequence for vertebrates was obtained from Hernández *et al*.[11]

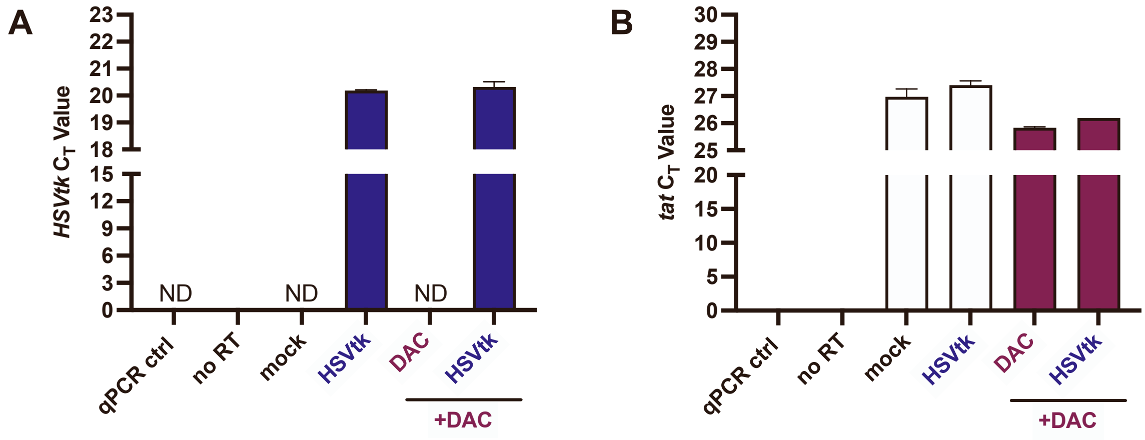

Figure S12

RT-qPCR analysis of LVV RNA payload and HIV-1 RNA target levels in J-Lat 10.6 cells ± DAC stimulation. 1 x 10^5 J-Lat 10.6 cells were seeded on day 1, stimulated with 25 nM DAC (or mock; media) on day 2, transduced with EF1α-directed LVVs (or mock; media) at an MOI of 14 on day 3, and lysed for RNA extraction on day 6. (**A**) Assay for LVV RNA payload based on *HSVtk* RT-qPCR on J-Lat 10.6 cellular RNA. (**B**) Assay for HIV-1 RNA target based on *tat* RT-qPCR on J-Lat 10.6 cellular RNA. (*A-B*) qPCR cycle threshold (C_T) values reported. Data presented as mean with SD (N = 2 qPCR replicates/condition).

SUPPLEMENTAL TABLES

Table S1

Primer sequences for mutagenesis PCRs.

Purpose	Primer Name	Sequence
**Mutate *BbV*CI site in CkRhsp-BD1-D4-pVAX-1**	Forward	5'- ATATTTTCCTATATTCTCCTCGCGGTGACGAAAACATGCTATCA-3'
	Reverse	5'-TGATAGCATGTTTTCGTCACCGCGAGGAGAATATAGGAAAATAT-3'
**Modify *HSVtk* internal ATGs – 1st round**	Forward	5'-GAAACTGCCCACGCTACTGCGGGTTTATATAGACGGTCCCCACGGGATCGGG-3'
	Reverse	5'-CCCGATCCCGTGGGGACCGTCTATATAAACCCGCAGTAGCGTGGGCAGTTTC-3'
**Modify *HSVtk* internal ATGs – 2nd round**	Tktr 3 Mut Forward	5'-ACGGTCCCCACGGGATCGGGAAAACCACCAC-3'
	Tktr 3 Mut Reverse	5'-GTGGTGGTTTTCCCGATCCCGTGGGGACCGT-3'
**Introduce *Xba*I to 5' end of *trans*-splicing cassette, pVAX-1 backbone**	5'*Xba*I Forward	5'-GACATTGATTATTGTCTAGAACTAGTTGAGCCCCACG-3'
	5'*Xba*I Reverse	5'-CGTGGGGCTCAACTAGTTCTAGACAATAATCAATGTC-3'
**Introduce *Xho*I upstream of poly(A) site in *trans*-splicing cassette, pVAX-1 backbone**	3'*Xho*I forward	5'-GGGAGGCGAACTGACTCGAGAACTTGTTTATTGC-3'
	3'*Xho*I reverse	5'-GCAATAAACAAGTTCTCGAGTCAGTTCGCCTCCC-3'
**Introduce *Xba*I and *Bcu*I to 5' end of EF1α promoter and *Nhe*I to 3' end**	EF1α Forward	5'-TCTAGAACTAGTGCTCCGGTGCCCGTCAG-3'
	EF1α Reverse	5'-GCTAGCTCACGACACCTGAAATGGAAG-3'

Table S2

Primer and probe sequences for PCR and sequencing.

Target	Primer or Probe Name	Sequence
AmpR	ampFP[12]	5’-GTTGCCATTGCTACAGGCATC-3’
	ampRP[12]	5’-ACTCGCCTTGATCGTTGGG-3’
	ampRP[12]	5’-[6FAM]ACGCTCGTCGTTTGGTATGGCTTCATTC[BHQ1]-3’
β-actin	β-actin Forward[13]	5’-GAGCGGTTCCGCTGCCCTGAGGCACTC-3’
	β-actin Reverse[13]	5′-GGGCAGTGATCTCCTTCTGCATCCTG-3′
hALB	HuALB intron 12 forward[14]	5′-GCTGTCATCTCTTGTGGGCTGT-3′
	HuALB intron 12 reverse[14]	5′-ACTCATGGGAGCTGCTGGTTC-3′
**HIV-1 Ψ**	233LentiP.F[14]	5’-CAGGACTCGGCTTGCTGAAG-3’
	363LentiP.R[14]	5′-TCCCCCGCTTAATACTGACG-3′
HSVtk	FP-HSV-uni amp[5]	5’-AAGCGCCCAGATAACAATGG-3’
	RP-HSV-uni amplicon[5]	5’-CCTCCCCCCCGATATGAG-3’
	HSV-uni amplicon probe[5]	5’-[6FAM]CATGCCTTATGCCGTGACCGACG[BHQ1]-3’
Tat	All-tat/vpr forward[15]	5’-TCCTATGGCAGGAAGAAGCG-3’
	All-tat/vpr reverse[15]	5’-AGCTTGATGAGTCTGACTGT-3’
	All-tat/vpr probe[15]	5’-[6FAM]TCTGATGAGCTCTTCGTCGCTGTCTC[TAM]-3’
***Trans*-spliced HIV-1 *tat-HSVtk* Short Amplicon**	All-tat/vpr forward[15]	5’-TCCTATGGCAGGAAGAAGCG-3’
	3’ ER Reverse Primer[16]	5’-CAGGGTTCTCCTCCACGTCTC-3’
***Trans*-spliced HIV-1 *tat-HSVtk* Long Amplicon**	BD D4 Forward	5’-GAGCCCTGGAAGCATCCAG-3’
	BD D4 Reverse	5’-CGCAGACGCGTGTTGATG-3’
WPRE	WPRE-forward[17]	5’-GGCACTGACAATTCCGTGGT-3’
	WPRE-reverse[17]	5’-AGGGACGTAGCAGAAGGACG-3’
	WPRE-probe[17]	5’-[6FAM]ACGTCCTTTCCATGGCTGCTCGC[TAM]-3’
**TOPO backbone**	M13 Forward (-20)[18]	5’-GTAAAACGACGGCCAG-3’
	M13 Reverse[18]	5´-CAGGAAACAGCTATGAC-3´

SUPPLEMENTAL METHODS

Karyotyping

Jurkat T cells were treated overnight with 1.43 µM thymidine (Sigma), after which cells were incubated in fresh media at 37°C for a further 4.5 hours. Cells were then treated with 0.1 µg/mL colcemid (Sigma) for 10 minutes at 37°C, pelleted, and resuspended in 0.055M KCl. After pelleting, three rounds of fixation were performed with 3:1 (*v/v*) methanol:glacial acetic acid. G-banded karyotyping was undertaken on fixed cells by the Cytogenetics Laboratory (Medical Genetics Service) at Cambridge University Hospitals.

Preparation of Therapeutic/Control LVV: Original Method

Two different methods were explored. In the original method (adapted from Dull *et al*[1] and Cribbs *et al*[19]), 5 x 10^6 HEK293T cells were plated in 10-cm-diameter dishes (one per LVV preparation) in the evening on day 1. To produce VSV-G-pseudotyped therapeutic/control LVVs, cells were transfected 24 hr later on day 2 as described in **Materials and Methods**. Media was changed the morning of day 3, and the following morning on day 4 lentiviral supernatants (~10 mL) were clarified from cellular debris (2103×*g*, 10 min) and combined with media to 30 mL for concentration by ultracentrifugation (described in **Materials and Methods**). LVV pellets were resuspended in PBS to an approximate volume of 1 mL. The original method was used solely for comparison against an optimized method (see **Materials and Methods**) used to prepare all LVV described in the text unless otherwise specified.

Estimation of Total LVV Particles by HIV-1 p24 Capsid ELISA

HIV-1 p24 capsid protein ELISAs were performed as described by Ingemarsdotter *et al*[20] using aliquots of LVV preparations that were diluted 1:100 in media and then inactivated in 0.1% Empigen for 30 min at 56°C. The final wash prior to incubation with Lumiphos Plus was performed with PBS instead of PBS-0.1% Tween 20.

An estimate for the number of LVV particles per mL was based on the observation that a single HIV-1 virion contains approximately 2000 Gag copies, associating 1 ng p24 (24 kDa) with approximately 1.25 x 10^7 LVV particles.[21]

Estimation of Plasmid Carryover by qPCR

The extent of lentiviral plasmid that co-purified with cellular DNA following transduction with LVV preparations was assessed with qPCR for *ampR,* with one copy present in LVV plasmids and none in the resultant LVV. Reactions were prepared in 1X Fast SYBR Green Master Mix or 1X TaqMan Fast Advanced Master Mix (Applied Biosystems) with 20-32 nM primer, 100 nM probe (for TaqMan reactions only) and 40-100 ng DNA template. A plasmid standard curve was prepared from 1:10 serial dilutions of pSico. *AmpR* copy number was divided by the estimate for cell number (*ALB* qPCR) to determine the average number of residual plasmid copies per cell following LVV transduction.

Estimation of Vector Copy Number by qPCR: Alternative Assay

Assays for HIV-1 or therapeutic/control LVV copies in cells transduced with lentivirus preparations were initially based on detection of the *psi* packaging signal (common to both HIV-1 and therapeutic/control LVVs) in cellular DNA, with qPCRs prepared in Fast SYBR Green Master Mix with 25 nM primer and 80-100 ng DNA template. A plasmid standard curve was prepared with pSico. To estimate the average vector copy number per cell (VCN) following lentiviral transduction, *psi* copy number was divided by the estimate for cell number (*ALB* qPCR).

Microscopy
Live cells were visualized with a widefield Nikon TE200 microscope at 10X magnification. An exposure of 10 ms was used for brightfield images. EGFP was excited at 490 nm (50% intensity) and exposures were taken at 1200 ms. Images were processed with ImageJ software.

SUPPLEMENTAL REFERENCES

1. Dull, T., Zufferey, R., Kelly, M., Mandel, R. J., Nguyen, M., Trono, D. & Naldini, L. A third-generation lentivirus vector with a conditional packaging system. *J. Virol.* **72**, 8463–71 (1998).
2. Zufferey, R., Dull, T., Mandel, R. J., Bukovsky, A., Quiroz, D., Naldini, L. & Trono, D. Self-inactivating lentivirus vector for safe and efficient in vivo gene delivery. *J. Virol.* **72**, 9873–80 (1998).
3. Farazmandfar, T., Haghshenas, M. R. & Shahbazi, M. Inhibition of HIV-1 by a Lentiviral Vector with a Novel Tat-Inducible Expression System and a Specific Tropism to the Target Cells. *Hum. Gene Ther.* **26**, 680–687 (2015).
4. Kim, D. W., Uetsuki, T., Kaziro, Y., Yamaguchi, N. & Sugano, S. Use of the human elongation factor 1 alpha promoter as a versatile and efficient expression system. *Gene* **91**, 217–223 (1990).
5. Poddar, S., Loh, P. S., Ooi, Z. H., Osman, F., Eul, J. & Patzel, V. RNA Structure Design Improves Activity and Specificity of trans-Splicing-Triggered Cell Death in a Suicide Gene Therapy Approach. *Mol. Ther. Nucleic Acids* **11**, 41–56 (2018).
6. Reese, M. G., Eeckman, F. H., Kulp, D. & Haussler, D. Improved splice site detection in Genie. *J. Comput. Biol.* **4**, 311–323 (1997).
7. Harrison, G. P., Miele, G., Hunter, E. & Lever, A. M. L. Functional analysis of the core human immunodeficiency virus type 1 packaging signal in a permissive cell line. *J. Virol.* **72**, 5886–5896 (1998).
8. Korneyeva, M., Stålhandske, P. & Asjö, B. Jurkat-tat but not other tat-expressing cell lines support replication of slow/low type HIV. *J Acquir Immune Defic Syndr* **6**, 231–6 (1993).
9. Chung, C.-H., Mele, A. R., Allen, A. G., Costello, R., Dampier, W., Nonnemacher, M. R. & Wigdahl, B. Integrated Human Immunodeficiency Virus Type 1 Sequence in J-Lat 10.6. *Microbiol. Resour. Announc.* **9**, e00179-20 (2020).
10. Sertznig, H., Hillebrand, F., Erkelenz, S., Schaal, H. & Widera, M. Behind the scenes of HIV-1 replication: Alternative splicing as the dependency factor on the quiet. *Virology* **516**, 176–188 (2018).
11. Hernández, G., Osnaya, V. G. & Pérez-Martínez, X. Conservation and Variability of the AUG Initiation Codon Context in Eukaryotes. *Trends Biochem. Sci.* **44**, 1009–1021 (2019).
12. Sastry, L., Xu, Y., Cooper, R., Pollok, K. & Cornetta, K. Evaluation of Plasmid DNA Removal from Lentiviral Vectors by Benzonase Treatment. *Hum. Gene Ther.* **15**, 221–226 (2004).
13. Duffy, S. & Cochrane, A. Analysis of HIV-1 RNA Splicing. in *Alternative pre-mRNA Splicing: Theory and Protocols* 6365–448 (Wiley-VCH Verlag GmbH & Co. KGaA, 2012).
14. Charrier, S., Dupré, L., Scaramuzza, S., Jeanson-Leh, L., Blundell, M. P., Danos, O., Cattaneo, F., Aiuti, A., Eckenberg, R., Thrasher, A. J., *et al.* Lentiviral vectors targeting WASp expression to hematopoietic cells, efficiently transduce and correct cells from WAS patients. *Gene Ther.* **14**, 415–428 (2007).
15. Norton, N. J., Mok, H. P., Sharif, F., Hirst, J. C. & Lever, A. M. L. HIV silencing and inducibility are heterogeneous and are affected by factors intrinsic to the virus. *MBio* **10**, e00188-19 (2019).
16. Ingemarsdotter, C. K., Poddar, S., Mercier, S., Patzel, V. & Lever, A. M. L. Expression of Herpes Simplex Virus Thymidine Kinase/Ganciclovir by RNA Trans-Splicing Induces Selective Killing of HIV-Producing Cells. *Mol. Ther. Nucleic Acids* **7**, 140–154 (2017).
17. Dalsgaard, T., Cecchi, C. R., Askou, A. L., Bak, R. O., Andersen, P. O., Hougaard, D., Jensen, T. G., Dagnæs-Hansen, F., Mikkelsen, J. G., Corydon, T. J., *et al.* Improved Lentiviral Gene

Delivery to Mouse Liver by Hydrodynamic Vector Injection through Tail Vein. *Mol. Ther. Nucleic Acids* **12**, 672–683 (2018).
18. Invitrogen. TOPO® TA Cloning® Kit. https://assets.thermofisher.com/TFS-Assets/LSG/manuals/topota_man.pdf.
19. Cribbs, A. P., Kennedy, A., Gregory, B. & Brennan, F. M. Simplified production and concentration of lentiviral vectors to achieve high transduction in primary human T cells. *BMC Biotechnol.* **13**, 98 (2013).
20. Ingemarsdotter, C. K., Zeng, J., Long, Z., Lever, A. M. L. & Kenyon, J. C. An RNA-binding compound that stabilizes the HIV-1 gRNA packaging signal structure and specifically blocks HIV-1 RNA encapsidation. *Retrovirology* **15**, 25 (2018).
21. Rein, A. RNA Packaging in HIV. *Trends Microbiol.* **27**, 715–723 (2019).
